# HbA1c-to-HDL-C ratio and risk of incident cardiovascular disease among older adults: evidence from a 7-year prospective cohort study in southwestern China

**DOI:** 10.3389/fpubh.2026.1901063

**Published:** 2026-09-17

**Authors:** Jin Peng, Liurong Yang, Qian Zhang, Xiangni Chen, Ying Tang, Na Xie

**Affiliations:** 1 Department of Chronic Disease Prevention and Control, Wanzhou District Center for Disease Control and Prevention, Chongqing, China; 2 Department of Healthcare-Associated Infection Control, Chongqing Academy of Medical Sciences, Chongqing General Hospital, Chongqing University, Chongqing, China

**Keywords:** cardiovascular disease, dose-response relationship, elderly, HbA1c/HDL-C ratio, prospective cohort, risk prediction

## Abstract

**Background:**

Cardiovascular disease (CVD) remains the leading cause of death worldwide, particularly among older adults. The glycated hemoglobin A1c-to-high-density lipoprotein cholesterol (HbA1c/HDL-C) ratio is an emerging biomarker reflecting glucolipid metabolic derangement. However, prospective evidence regarding its association with incident CVD among community-dwelling older adults in southwestern China remains limited.

**Objective:**

This study aimed to investigate the association between the HbA1c/HDL-C ratio and incident CVD, characterize its dose–response relationship, and evaluate its incremental predictive value beyond conventional cardiovascular risk factors in an older adult population.

**Methods:**

This prospective cohort study included 1,875 community-dwelling adults aged ≥60 years without baseline CVD from the Early Screening and Intervention Program for High-Risk Populations of Cardiovascular and Cerebrovascular Disease Risk Factors in Wanzhou District. Participants were followed during 2024. The HbA1c/HDL-C ratio was calculated from baseline measurements and analyzed both continuously and as quartiles. Incident CVD events were ascertained using a dual-source validation approach that combined standardized questionnaire data with linkage to the regional surveillance system. Multivariable Cox proportional hazards models, restricted cubic spline (RCS), time-dependent area under the receiver operating characteristic curve (AUC), C-statistic and decision curve analysis (DCA) were fitted to estimate the association between HbA1c/HDL-C and CVD risk. Subgroup, sensitivity analyses were performed to verify robustness.

**Results:**

During a median follow-up of 5.08 years, 207 participants developed incident CVD, with a cumulative incidence of 11.04%. After fully adjusting for confounding factors, participants in the highest quartile (Q4) of the HbA1c/HDL-C ratio exhibited a significantly elevated risk of incident CVD compared with the lowest quartile (Q1) (HR = 2.111, 95%*CI*: 1.276–3.170, *P* = 0.003), whereas the linear trend across quartiles did not reach statistical significance (*P*_for trend_ = 0.068). RCS analysis revealed a nonlinear dose-response relationship (*P*_for overall_ < 0.001, *P*_for nonlinear_ = 0.017), with a data-driven inflection point at an HbA1c/HDL-C ratio of 4.83 (95%*CI*: 3.55–6.06). Below this threshold, each unit increment was associated with a 44.10% increase in CVD risk (HR = 1.441, 95%*CI*: 1.138–1.825, *P* = 0.002). Incorporation of the HbA1c/HDL-C ratio into a conventional risk factor model enhanced the time-dependent AUC (test-set C-statistic: 0.795 vs. 0.777). DCA demonstrated enhanced net benefit, particularly during mid-to-long-term follow-up. Subgroup and sensitivity analyses verified the robustness.

**Conclusions:**

After multivariable Cox regression adjustment, participants in the highest quartile (Q4) of the HbA1c/HDL-C ratio showed a significant association with incident CVD, while the linear trend across Q1 to Q4 was borderline significant. RCS analysis further identified a nonlinear dose–response relationship between HbA1c/HDL-C ratio and incident CVD. Given its accessibility, low cost, and ease of calculation, the HbA1c/HDL-C ratio may serve as a complementary indicator for community-based CVD risk stratification, particularly in resource-constrained primary care settings. The apparently stronger association observed should be considered exploratory and requires confirmation in larger prospective cohort studies.

## Introduction

Cardiovascular disease (CVD) remains the leading cause of death and disability worldwide. Data from the Global Burden of Disease Study 2021 indicated that CVD caused approximately 9.42 million deaths in 2021, representing a 57.5% increase relative to 1991 (1). The burden of CVD is disproportionately borne by low and middle-income countries, where rapid epidemiological transitions have accelerated the prevalence of cardiovascular risk factors and disease (1). China is facing a growing burden of CVD, driven by population aging, urbanization, and unhealthy lifestyle changes and the rising prevalence of metabolic risk factors. The 2022 Report on Cardiovascular Health and Diseases in China estimated that approximately 330 million people in China were living with CVD, including 245 million with hypertension, 13 million with stroke, and 11.39 million with coronary heart disease (2). National cohort projections indicated that the annual incidence of CVD in China was expected to rise from 0.74% in 2021 to 0.97% by 2030.

Although substantial progress had been made in community-based prevention and access to inpatient healthcare services, CVD mortality is projected to stabilize only after 2024, underscoring the persistent public health challenge posed by cardiovascular disease (3). In 2021, the burden of CVD among older adults in China increased steeply with age. Among individuals aged ≥65 years, the mortality rate rose from 644.87 per 100,000 population in the 65–69-year age group to 14,370.42 per 100,000 in the 90–94-year age group. A similar age-related gradient was observed for DALYs, which increased from 17,225.45 to 128,096.14 per 100,000 population across the same age groups (4). These data highlight the urgent need for effective primary prevention strategies targeting older adults, who experience the highest burden of cardiovascular morbidity and mortality.

Traditional risk factors such as hypertension, dyslipidemia, diabetes, smoking, and obesity were well-established determinants of CVD (5), existing risk prediction models remain suboptimal in older adults. Although the Framingham Risk Score has served as the cornerstone of CVD risk prediction for decades, its ability to discriminate risk diminishes substantially among adults aged >70 years. This decline reflected the disproportionate contribution of chronological age to risk estimation and the weakened prognostic relevance of traditional cardiovascular risk factors in older populations (6, 7). Similarly, the European SCORE system was designed primarily for middle-aged adults and had shown limited predictive accuracy among older individuals, thereby restricting its applicability in geriatric populations (8).

The limitations of conventional risk prediction models have prompted increasing investigation into novel biomarkers that reflect residual metabolic risk. Composite indicators, such as the triglyceride-glucose (TyG) index (9), the atherogenic index of plasma (AIP) (10), and glycemic–lipid interaction metrics, have demonstrated potential for improving CVD risk prediction beyond traditional cardiovascular risk factors (11). Nevertheless, evidence regarding the predictive value of these emerging biomarkers in community-dwelling older adults remains scarce. Most studies have been conducted in middle-aged populations or selected high-risk clinical cohorts, leaving an important knowledge gap, particularly in rural southwestern China, where constrained healthcare resources underscore the need for simple and accessible risk assessment tools.

The HbA1c/HDL-C ratio, a composite index integrating the glycemic marker HbA1c and the lipid indicator HDL-C, has attracted considerable research attention. Emerging evidence from the China Health and Retirement Longitudinal Study (CHARLS) suggested that elevated HbA1c-to-HDL-C levels correlated with increased likelihoods of stroke, advancing carotid atherosclerosis, and multiple cardiometabolic conditions among aging adults in China (12–14). Furthermore, retrospective evidence indicated significant associations of HbA1c-to-HDL-C with coronary artery calcification were observed in asymptomatic adults (15). However, there is a paucity of prospective cohort studies in rural Chinese elderly populations evaluating the long-term predictive ability of HbA1c/HDL-C ratio for incident CVD events, particularly with regard to effect modification by metabolic health status. This gap in evidence limits its translation into primary care practice and public health decision-making, especially in resource-constrained settings where comprehensive cardiovascular risk assessment is difficult.

To address these gaps, we leveraged data from the Early Screening and Intervention Program for High-Risk Populations of Cardiovascular and Cerebrovascular Disease Risk Factors in Wanzhou District, a well-established community-based cohort with standardized biennial assessments and rigorous outcome ascertainment. The current study aimed to: (1) examine the relationship between HbA1c/HDL-C ratio and incident CVD among older adults during 7-year follow up; (2) characterize the dose–response relationship using restricted cubic spline analysis; (3) quantify the additional predictive capacity of the HbA1c-to-HDL-C ratio regarding CVD events; and (4) explore effect modification across clinical subgroups, thereby providing new evidence to inform CVD prevention and control in southwestern China. To our knowledge, this is among the first prospective studies to investigate the association between the HbA1c/HDL-C ratio and incident CVD among rural older adults in southwestern China, thereby providing region-specific evidence to inform local CVD prevention strategies.

## Method

### Data source and study population

All data were derived from the Early Screening and Intervention Program for High-Risk Populations of Cardiovascular and Cerebrovascular Disease Risk Factors in Wanzhou District. Baseline data were collected in 2017, and follow-up was conducted from January to December 2024 (end date: December 31, 2024), yielding a 7.0-year follow-up period. Written informed consent was obtained from all participants. Ethical approval was granted by the Wanzhou District Center for Disease Control and Prevention (No. WZJK-00215). This study adhered to the STROBE guidelines for reporting observational studies (16).

For the current analysis, we utilized the 2017 baseline data, with follow-up conducted in 2024 to ascertain study outcomes. To maintain data validity and study rigor, the exclusion criteria outlined below were applied: (1) participants younger than 60 years in 2017; (2) participants with missing CVD data or a pre-existing CVD diagnosis in 2017; (3) participants with absent baseline data on HbA1c, HDL-C, or any covariate; and (4) participants lost to follow-up or with missing CVD outcome data from 2017 to 2024. A total of 3,417 individuals met the initial screening criteria for follow-up during the study period. After follow-up, a total of 244 incident CVD cases were identified. Among participants aged ≥60 years at baseline screening, 207 developed CVD, accounting for 84.84% of all cases (Supplementary Table 1). After applying the predefined exclusion criteria, 1,875 participants were included in the final analysis (Figure 1). The comparison of baseline characteristics between included and excluded participants were presented in Supplementary Table 1. Over the follow-up period, a total of 26 deaths occurred, of which 6 (23.1%) were attributed to cardiovascular disease.

**Figure 1:**
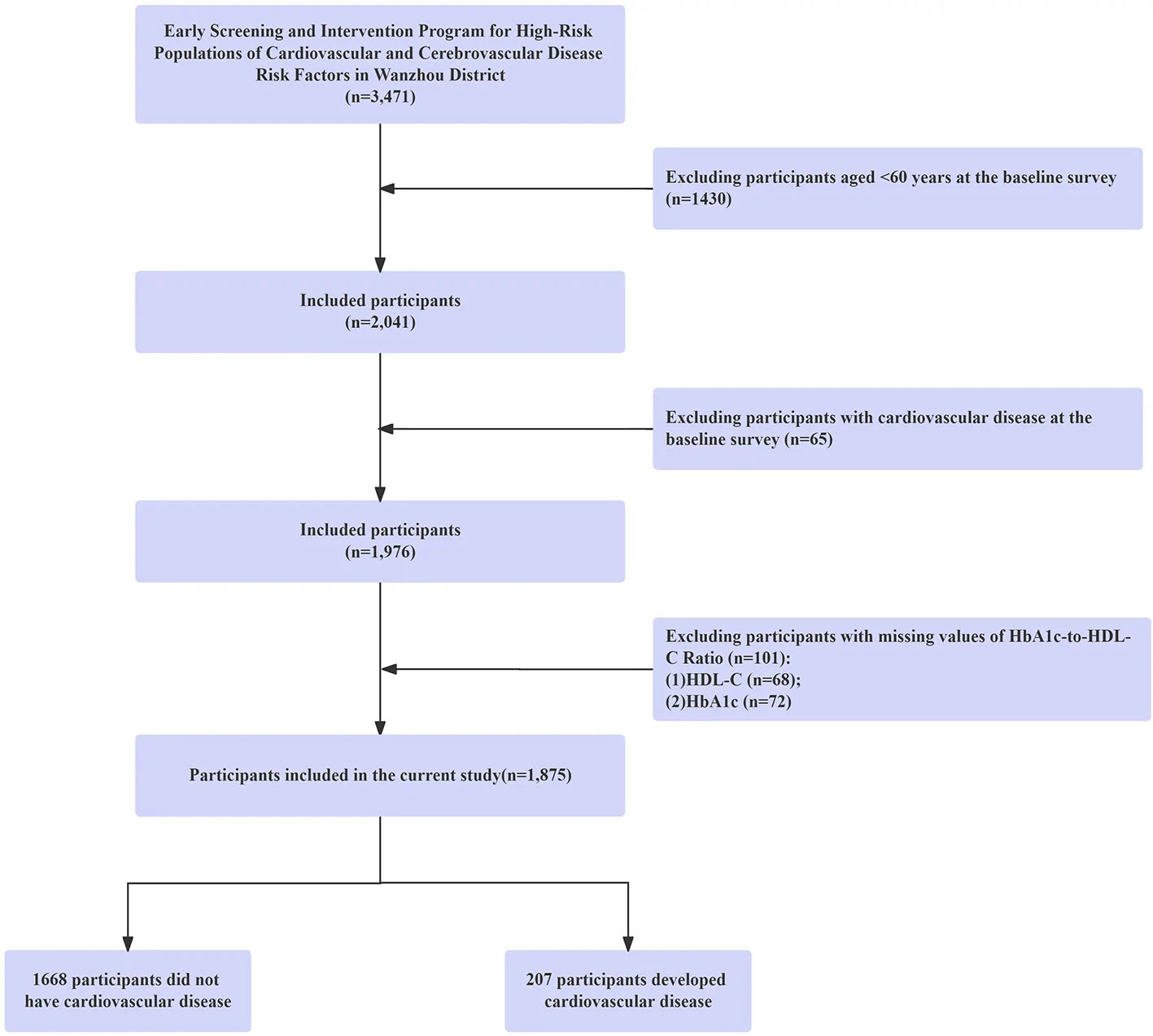
Flowchart of the study participants selection process. HDL-C, high-density lipoprotein cholesterol; HbA1c, hemoglobin A1c.

### Data collection and measurement

A computer-assisted personal interviewing (CAPI) system was used to collect standardized information on demographic characteristics, health status, lifestyle behaviors, and socioeconomic conditions. All interviewers were systematically trained and certified competent through standardized assessments administered by the Wanzhou District Center for Disease Control and Prevention (WanZhou CDC). Data were obtained through face-to-face interviews conducted by trained personnel.

During the baseline survey and follow-up examinations, physical measurements and laboratory assessments were performed, including height, weight, blood pressure, fasting blood glucose, glycated hemoglobin (HbA1c), and lipid profile parameters. Blood samples were collected by experienced laboratory technicians with the rank of associate chief physician or above. Participants were instructed to fast for at least 8 h prior to venipuncture. Trained personnel collected venous blood samples and conducted biochemical measurements in accordance with standardized operating procedures. Specimens were transported under cold-chain conditions and analyzed centrally at Chongqing University Three Gorges Hospital. Biochemical assays included triglycerides (TG), total cholesterol (TC), low-density lipoprotein cholesterol (LDL-C), and high-density lipoprotein cholesterol (HDL-C), which were measured using enzymatic colorimetric methods, while HbA1c was quantified using boronate affinity high-performance liquid chromatography. All test results were independently reviewed and cross-verified by two designated staff members at the Wanzhou CDC to ensure accuracy and inter-rater reliability. Anthropometric measurements were performed using standardized procedures. Height was measured in the erect position (heels together, weight evenly distributed) by a stadiometer. Body weight was recorded on a calibrated digital scale with participants in light clothing and barefoot.

### Definition of the HbA1c/HDL-C ratio

The HbA1c/HDL-C ratio is calculated as glycated hemoglobin (HbA1c, %) divided by high-density lipoprotein cholesterol (HDL-C, mmol/L) using measurements obtained at the 2017 baseline survey only, by the following formula (17),


$$\begin{array}{l} HbA1c/HDL\;-C\;ratio=\frac{HbA1c\;\left(\%\right)}{HDL-C\;\left(\frac{mmol}{L}\right)} \end{array}$$

consistent with the CHARLS study in China. The HbA1c/HDL-C ratio was categorized into quartiles: Q1 (0.98–2.77), Q2 (2.78–3.46), Q3 (3.47–4.33), and Q4 (≥4.34) for further analyses. In Cox models, the ratio was analyzed as a categorical variable with Q1 as the reference, and as a continuous variable in restricted cubic spline (RCS) analyses.

### Assessment of cardiovascular disease events

Primary incident CVD outcomes were defined as the first cardiovascular event occurring during follow-up. Outcome ascertainment was based on a dual-source validation process. First, self-reported physician-diagnosed CVD was obtained using a standardized questionnaire based on the international classification of diseases-10 edition, including coronary heart disease (angina pectoris [I20]; myocardial infarction [I21–I22]; asymptomatic coronary stenosis [I24]), atrial fibrillation (I48), valvular heart disease (I05–I09, I34–I39), and other cardiac disorders. Second, all incident CVD cases occurring between 2017 and 2024 were further verified through linkage with the Wanzhou Cardiovascular and Cerebrovascular Disease Surveillance System, which only recorded three categories of CVD: acute myocardial infarction (I21), subsequent myocardial infarction (I22), and sudden cardiac death (I46.1). All self-reported myocardial infarction events were verified through the regional surveillance system, and an additional five incident myocardial infarction cases were identified through registry records. However, self-reported angina pectoris, asymptomatic coronary stenosis, atrial fibrillation, and valvular heart disease were not routinely captured by the surveillance system. Therefore, the ascertainment of these conditions relied solely on participants' self-reported information. Stroke, heart failure, peripheral artery disease, and coronary revascularization were not included in the primary composite endpoint. Consequently, all participants included in the present study were free of prevalent CVD at baseline. During follow-up, incident CVD, CVD-specific death, and death from other causes were recorded as distinct outcomes.

Due to system upgrades and other technical adjustments, we implemented a multi-channel participant tracing strategy prior to the formal follow-up. First, participants were contacted by telephone to confirm their follow-up time and location. For individuals who could not be reached using their primary telephone numbers, alternative contact information was obtained from the resident health record system and the community livelihood management platform. For participants who remained unreachable after repeated telephone attempts, community staff conducted home visits to notify them of the follow-up arrangements. Consequently, only nine participants were ultimately lost to follow-up. These individuals were permanently removed from the continuous study database and excluded from all analytical datasets. Accordingly, neither their baseline characteristics nor follow-up outcome data were included in any statistical analyses. The predefined endpoint cessation criteria were as follows: the occurrence of incident CVD, all-cause mortality, or administrative censoring at the end of the follow-up period in the absence of incident CVD events.

Event time was determined as the duration from baseline examination to the first occurrence of CVD. For participants who remained free of CVD throughout the observation period, follow-up was censored at the last follow-up visit, event time was defined as the interval between the date of last follow-up time and the baseline survey date. For participants who died from CVD, the event time was defined as the interval between the date of incident CVD diagnosis and the baseline survey date. For those who died from non-cardiovascular causes, follow-up was censored at the date of death, the event time was calculated as the interval between the date of death and the baseline survey date. Information on deceased participants, including the date and cause of death, was obtained from their family members through standardized follow-up interviews. For participants who died from CVD, the date of CVD onset was additionally obtained from their relatives.

### Covariates

The selection of covariates was guided by prior epidemiological evidence and and directed acyclic graph (DAG) analysis (Supplementary Figure 1), limited to variables available within the study database (18). Baseline sociodemographic characteristics were collected via structured questionnaires, including gender (males, females), age (categorized as 60–64 years, 65–69 years, 70–74 years, ≥75 years), marital status (categorized as separated/divorced/widowed/unmarried or married/cohabiting), education level (categorized as primary school and below, junior middle school, technical secondary/high school, college and above), job (categorized as others and farmers), residential status (categorized as living alone, living only with spouse, living only with children, living with spouse and children) and annual personal income (categorized as < 4,999 yuan, 5,000–9,999 yuan, 10,000–19,999 yuan, ≥20 000 yuan. Additionally, health-related and lifestyle behaviors comprised smoking status (No, Yes), drinking status (categorized as non-drinker, light drinking, heavy drinking regularly), exercise habits (categorized as regular exercise, insufficient exercise), dietary taste (categorized as moderate, light taste, salty taste), dietary habits (categorized as balanced diet, vegetarian-biased diet, meat-biased diet, vegetable and fruit consumption frequency (categorized as ≤ 2 days per week, 3–4 days per week, ≥5 days per week). Physiological parameters were assessed through standardized clinical examinations, including body weight, height, systolic blood pressure (SBP), and diastolic blood pressure (DBP). Laboratory analyses comprised TC, TG, LDL-C, HDL-C, blood glucose and HbA1c.

Stringent quality control procedures were implemented at baseline to minimize missing data. The data management system was programmed to prevent the storage and submission of incomplete questionnaires, thereby ensuring complete questionnaire data for all enrolled participants. Standardized protocols were strictly followed throughout blood sample collection and biochemical laboratory testing. Participants who completed the questionnaire but were unable to provide blood samples were excluded during enrollment. All covariates included in the multivariable models were complete for the 1,875 participants included in the final analytical sample; therefore, no data imputation was required. The completeness rate for all covariates was 100% in the analytical sample.

The diagnosis of hypertension (19) was operationalized based on the following criteria: SBP≥140 mmHg and/or DBP≥90 mmHg, documented physician diagnosis via self-report, or current engagement in antihypertensive medication or lifestyle therapy for blood pressure control. Diabetes (20) was defined as FPG≥7.0 mmol/L or/and HbA1c≥6.50%, a self-reported clinical diagnosis, or current treatment with glucose-lowering medication or prescribed lifestyle intervention. Dyslipidemia (21) was defined as the presence of at least one of the following lipid abnormalities: TC≥6.2 mmol/L, TG≥2.3 mmol/L, LDL-C≥4.1 mmol/L, or HDL-C < 1.0 mmol/L. Body mass index (BMI) was calculated as weight in kilograms divided by the square of height in meters (kg/m^2^) (22). Consistent with Chinese adult criteria, participants were categorized as overweight if their BMI was 24.0–27.9 kg/m^2^ and as obese if their BMI was≥28.0 kg/m^2^ (22).

### Statistical analysis

The distribution of continuous variables was evaluated using the Shapiro–Wilk test. Normally distributed variables are presented as mean ± SD and were compared using independent-samples *t*-tests, whereas non-normally distributed variables are reported as median (IQR) and were compared using the Mann–Whitney U test. Categorical variables are presented as frequencies and percentages and were compared using the chi-square test or Fisher's exact test, as appropriate. Because no established clinical threshold exists for the HbA1c/HDL-C ratio, this variable was examined continuously and as quartiles in subsequent analyses.

The association between the HbA1c/HDL-C ratio and incident CVD was evaluated using multivariable Cox proportional hazards regression models. To minimize potential overadjustment arising from the inclusion of variables on the causal pathway, we adopted a hierarchical modeling strategy with progressive covariate adjustment. Accordingly, six models with sequential adjustment for potential confounders were constructed: Model 1, unadjusted. Model 2, adjusted for demographic characteristics (sex, age at screening, marital status, educational level, job, residential status, and annual personal income). Model 3, further adjusted for clinical variables (family history of coronary heart disease, family history of hypertension, family history of diabetes). Model 4, additionally adjusted for lifestyle factors (smoking status, drinking status, exercise habits, dietary taste, dietary patterns, vegetable consumption frequency, and fruit consumption frequency), which indicated the total association between HbA1c/HDL-C ratio and incident CVD. Model 5, additionally adjusted for hypertension and overweight/obesity and Model 6, further adjusted for diabetes, dyslipidemia. Model 6 estimated the association independent of established metabolic diseases and conventional risk factors. Given that Model 6 included a large number of covariates, overadjustment bias may have been introduced. Therefore, Model 6^a^ was constructed based on Model 6 and included only variables that were statistically significant in the univariate analyses (*P* < 0.05). Collinearity diagnostics were performed for all variables included in the multivariable Cox regression models. Variance inflation factor (VIF) values below 5 indicated the absence of significant multicollinearity. We evaluated the proportional hazards assumption using Schoenfeld residuals (23).

To compare the associations of the HbA1c/HDL-C ratio and its individual components with incident CVD, we fitted additional Cox regression models based on Model 5 by separately incorporating continuous HbA1c, continuous HDL-C, or the continuous HbA1c/HDL-C ratio. The associations estimated from the fully adjusted models were then compared between: (1) Model 5 plus HbA1c or HDL-C entered separately; and (2) Model 5 plus the HbA1c/HDL-C ratio. This comparison allowed us to determine whether the composite ratio provided additional predictive information beyond its individual components.

Within the context of multivariable Cox proportional hazards regression, restricted cubic spline (RCS) modeling was implemented to explore potential nonlinear relationships between HbA1c/HDL-C and the incidence of CVD events. Knots were evenly allocated according to the quantile distribution of the HbA1c/HDL-C ratio, placed at the 5th, 35th, 65th, and 95th percentiles. After fitting the spline model, the inflection point, defined as the value at which the slope of the fitted curve changed substantially, was automatically identified. Hazard ratios (HRs) and corresponding 95% confidence intervals (CIs) were estimated separately for participants below and above the inflection point. The predicted risk of incident CVD across the full range of HbA1c/HDL-C ratio values was visualized using the RCS curve. The significance of the overall association and nonlinear relationship was evaluated using likelihood ratio tests. A *P* value for overall < 0.05 (*P*_for overall_ < 0.05) indicated a statistically significant relationship between HbA1c/HDL-C ratio and incident CVD, whereas a *P* value for nonlinear < 0.05 (*P*_for nonlinear_ < 0.05) indicated evidence of a nonlinear dose–response relationship.

To comprehensively evaluate the predictive value of the HbA1c/HDL-C ratio for incident CVD, we developed prognostic prediction models based on Cox proportional hazards regression. The study population was randomly divided into a training set (70%) and a test set (30%), with a fixed random seed to ensure reproducibility. Two models were constructed for comparison. Model A was the conventional clinical prediction model incorporating the same covariates as Model 6, whereas Model B was developed by additionally including the HbA1c/HDL-C ratio in Model A. Time-dependent area under the receiver operating characteristic curve (AUC) values and C-statistics were calculated at different follow-up time points to evaluate model discrimination, and corresponding 95% confidence intervals (CIs) were estimated.

Model discrimination was evaluated using two complementary metrics: (1) the time-dependent AUC, which assesses the model's ability to discriminate between participants who do and do not experience the event at specific event time points (e.g., 2, 3, 4, 5, 6, and 7 years), thereby capturing the dynamic changes in predictive performance over time; and (2) the concordance statistic (C-statistic), which represents the probability that, for a randomly selected pair of participants, the individual with the higher predicted risk experiences the event earlier and provides an overall measure of discriminative ability throughout the follow-up period. The time-dependent AUC characterizes discrimination at specific follow-up durations, whereas the C-statistic summarizes the model's overall discriminative performance across the entire follow-up period. Time-dependent AUC values at each follow-up time point and the overall C-statistic were calculated with their corresponding 95% confidence intervals (CIs).

Furthermore, decision curve analysis (DCA) was performed at multiple follow-up time points to evaluate the clinical utility of the prediction models by estimating the net benefit across a range of threshold probabilities, with net benefit serving as the primary evaluation metric. Optimized DCA curves were generated separately for the training and test sets to assess the clinical applicability and generalizability of the two models. DCA generated three curves, including (1) Model curve: the curve of the prognostic prediction model constructed in this study, which reflects the net benefit yielded by individual risk stratification using the established model across varying risk thresholds; (2) All curve: the reference line assuming all participants are classified as high-risk individuals and receive uniform risk alerts. Its net benefit decreases linearly as the risk threshold rises; (3) None curve: the reference line representing a strategy with no risk stratification or alert implementation for any participant, with a constant net clinical benefit of zero across all thresholds. Additionally, model calibration was evaluated at the 7-year follow-up using calibration plots, in which the predicted risk of CVD was compared with the observed incidence estimated by the Kaplan-Meier method. Model goodness of fit was further assessed using the Hosmer–Lemeshow test, with a *P* value > 0.05 indicating acceptable calibration.

To determine whether the HbA1c/HDL-C ratio provides incremental predictive value beyond existing metabolic indices, we compared its predictive performance with that of the following indices: (1) Fasting blood glucose (FBG); (2) HbA1c alone; (3) HDL-C alone; (4) HbA1c/HDL-C ratio; (5) The triglyceride-glucose (TyG) index, calculated as ln[TG (mg/dL) × fasting plasma glucose (mg/dL)/2] (24); (6) TG/HDL-C ratio; (7) non-HDL-C (total cholesterol minus HDL-C); and (8) the atherogenic index of plasma (AIP), calculated as log[TG (mmol/L)/HDL-C (mmol/L)] (25). As exploratory analyses, only multivariable Cox proportional hazards regression and receiver operating characteristic (ROC) curve analysis were performed. Each metabolic index was evaluated as a continuous variable in the fully adjusted model (Model 6). In addition, ROC curve analysis was conducted to compare the predictive performance of the individual indices.

Subgroup and sensitivity analyses were undertaken to verify the robustness of the main results. Subgroup analyses were performed across strata of CVD events, mainly including angina pectoris, myocardial infarction, atrial fibrillation, considering the limited CVD events. Primary association analyses were conducted using Cox proportional hazards regression models. Additionally, subgroup analyses were performed across strata of variables that were significantly associated with CVD risk to examine whether the association between the HbA1c/HDL-C ratio and incident CVD differed across demographic and clinical subgroups. Interaction effects were assessed using likelihood ratio tests.

Three sensitivity analyses were performed to verify the robustness of the primary study findings. First, the Fine-Gray competing risk model was applied to evaluate the associations of HbA1c/HDL-C ratio with mortality, treating death as a competing risk. Two hierarchical regression models were established for outcome assessment: Model 7 (unadjusted crude model) and Model 8 (fully adjusted model, with covariates consistent with those adopted in Model 6). Subdistribution hazard ratios (sHRs) and corresponding 95% confidence intervals (CIs) were calculated to quantify the effect sizes. Additionally, cumulative incidence functions (CIFs) were determined and presented in the analysis. Second, to estimate the total association unmediated by metabolic diseases, we excluded participants with HbA1c≥6.50% or/and HDL-C < 1.0 mmol/L in the sensitivity analyses, with Cox proportional hazards regression (Model 9), Kaplan-Meier cumulative hazard curves and RCS analyses were then repeated using the fully adjusted model (Model 6). Third, We also constructed Model 10, which excluded participants with hypertension, diabetes, dyslipidemia, and overweight/obesity for sensitivity analysis. Fourth, of the initial 3,471 participants, 1,596 individuals aged < 60 years were excluded, leaving 1,875 participants for the final analysis. This exclusion may have introduced selection bias. Therefore, we conducted supplementary Cox proportional hazards regression analyses among the excluded participants to examine the association between the HbA1c/HDL-C ratio and incident CVD. All statistical analyses were performed using R software (version 4.3.2). A two-sided *P*-value < 0.05 was considered statistically significant.

## Results

### Baseline characteristics

A total of 1,875 participants were enrolled in this study. The cohort had a mean age of 71.89 years, with females comprising 51.31%. The median follow-up duration of current study was 5.08 years (IQR: 3.57–5.19 years). By the end of follow-up, all-cause mortality had occurred in 26 participants (1.39%), of whom 6 (0.32%) died of CVD. We recorded 207 incident CVD events, yielding a cumulative incidence of 11.04%. The distribution of subtypes of CVD was presented in Supplementary Table 2, among all CVD cases, angina pectoris was the most prevalent subtype, accounting for 86 cases (41.55%). Compared with participants who remained free of CVD, those who developed CVD were older, more likely to be married or cohabiting, higher education and reported higher annual personal income. Furthermore, they exhibited a greater prevalence of hypertension, diabetes, dyslipidemia, and overweight/obesity, as well as family history of coronary heart disease, hypertension, and diabetes. Participants with incident CVD also demonstrated greater meat consumption, reduced frequencies of vegetable and fruit intake, and elevated HbA1c/HDL-C ratios (Table 1).

**Table 1:** Baseline characteristics of the study participants.

Variables	Total (*n* = 1,875)	Non-CVD (*n* = 1,668)	CVD (*n* = 207)	Statistic	*P*
Gender				0.001	0.976
Males	913 (48.69)	812 (88.94)	101 (11.06)		
Females	962 (51.31)	856 (88.98)	106 (11.02)		
Age at screening, years				23.793	< 0.001
60–64	282 (15.04)	256 (90.78)	26 (9.22)		
65–69	419 (22.35)	381 (90.93)	38 (9.07)		
70–74	531 (28.32)	490 (92.28)	41 (7.72)		
≥75	643 (34.29)	541 (84.14)	102 (15.86)		
Marital status				6.549	0.010
Separated/divorced/widowed/unmarried	270 (14.40)	228 (84.44)	42 (15.56)		
Married/cohabiting	1605 (85.60)	1440 (89.72)	165 (10.28)		
Educational level				30.105	< 0.001
Primary school and below	1163 (62.03)	1052 (90.46)	111 (9.54)		
Junior middle school	499 (26.61)	448 (89.78)	51 (10.22)		
Technical secondary/high school	150 (8.00)	123 (82.00)	27 (18.00)		
College and above	63 (3.36)	45 (71.43)	18 (28.57)		
Job				46.645	< 0.001
Others	225 (21.00)	172 (76.44)	53 (23.55)		
Farmers	1650 (88.00)	1496 (90.67)	154 (9.33)		
Residential status				7.517	0.057
Living alone	215 (11.47)	182 (84.65)	33 (15.35)		
Living only with spouse	1074 (57.28)	961 (89.48)	113 (10.52)		
Living only with children	114 (6.08)	97 (85.09)	17 (14.91)		
Living with spouse and children	472 (25.17)	428 (90.68)	44 (9.32)		
Annual personal income, yuan				33.544	< 0.001
<*4,999*	723 (38.56)	665 (91.98)	58 (8.02)		
5,000–9,999	421 (22.45)	385 (91.45)	36 (8.55)		
10,000–19,999	206 (10.99)	186 (90.29)	20 (9.71)		
≥20 000	525 (28.00)	432 (82.29)	93 (17.71)		
Family history of coronary heart disease				21.200	< 0.001
No	1764 (94.08)	1584 (89.80)	180 (10.20)		
Yes	111 (5.92)	84 (75.68)	27 (24.32)		
Family history of hypertension				16.732	< 0.001
No	1527 (81.44)	1380 (90.37)	147 (9.63)		
Yes	348 (18.56)	288 (82.76)	60 (17.24)		
Family history of diabetes				5.228	0.022
No	1761 (93.92)	1574 (89.38)	187 (10.62)		
Yes	114 (6.08)	94 (82.46)	20 (17.54)		
Hypertension				6.948	0.008
No	822 (43.84)	749 (91.12)	73 (8.88)		
Yes	1053 (56.16)	919 (87.27)	134 (12.73)		
Diabetes				16.666	< 0.001
No	1621 (86.45)	1461 (90.13)	160 (9.87)		
Yes	254 (13.55)	207 (81.50)	47 (18.50)		
Dyslipidemia				6.350	0.012
No	1319 (70.35)	1189 (90.14)	130 (9.86)		
Yes	556 (29.65)	479 (86.15)	77 (13.85)		
Overweight/obesity				7.265	0.007
No	1140 (60.80)	1032 (90.53)	108 (9.47)		
Yes	735 (39.20)	636 (86.53)	99 (13.47)		
Smoking status				0.050	0.823
No	1586 (84.59)	1412 (89.03)	174 (10.97)		
Yes	289 (15.41)	256 (88.58)	33 (11.42)		
Drinking status				4.805	0.090
Non-drinker	1641 (87.52)	1467 (89.40)	174 (10.60)		
Light drinking	184 (9.81)	161 (87.50)	23 (12.50)		
Heavy drinking regularly	50 (2.67)	40 (80.00)	10 (20.00)		
Exercise habits				0.046	0.830
Regular exercise	1514 (80.75)	1348 (89.04)	166 (10.96)		
Insufficient exercise	361 (19.25)	320 (88.64)	41 (11.36)		
Dietary taste				0.988	0.610
Moderate	1473 (78.56)	1308 (88.80)	165 (11.20)		
Light taste	274 (14.61)	248 (90.51)	26 (9.49)		
Salty taste	128 (6.83)	112 (87.50)	16 (12.50)		
Dietary habits				6.758	0.034
Balanced diet	1569 (83.68)	1391 (88.66)	178 (11.34)		
Vegetarian-biased diet	287 (15.31)	263 (91.64)	24 (8.36)		
Meat-biased diet	19 (1.01)	14 (73.68)	5 (26.32)		
Vegetable consumption frequency				10.115	0.006
≤ 2 days per week	1413 (75.36)	1239 (87.69)	174 (12.31)		
3–4 days per week	315 (16.80)	290 (92.06)	25 (7.94)		
≥5 days per week	147 (7.84)	139 (94.56)	8 (5.44)		
Fruit consumption frequency				58.385	< 0.001
≤ 2 days per week	496 (26.45)	397 (80.04)	99 (19.96)		
3–4 days per week	269 (14.35)	239 (88.85)	30 (11.15)		
≥5 days per week	1110 (59.20)	1032 (92.97)	78 (7.03)		
HbA1c/HDL-C ratio level				31.683	< 0.001
Q1	469 (25.01)	431 (91.90)	38 (8.10)		
Q2	473 (25.23)	441 (93.23)	32 (6.77)		
Q3	469 (25.01)	412 (87.85)	57 (12.15)		
Q4	464 (24.75)	384 (82.76)	80 (17.24)		

Q1, Quartile 1; Q2, Quartile 2; Q3, Quartile 3; Q4, Quartile 4; CVD, cardiovascular disease; HDL-C, high density lipoprotein; HbA1c, hemoglobin A1c.

The median HbA1c/HDL-C ratio was 3.46. Quartile-based stratification of HbA1c/HDL-C ratio revealed a different distribution in variables of gender, educational level, job, annual personal income, family history of coronary heart disease, family history of hypertension, family history of diabetes, hypertension, diabetes, dyslipidemia, overweight/obesity, smoking status, fruit consumption frequency (Supplementary Table 3). With respect to continuous metabolic parameters, participants who developed CVD exhibited higher BMI, HbA1c, total cholesterol, LDL-C, and HbA1c/HDL-C ratio levels, whereas those who remained CVD-free demonstrated elevated HDL-C concentrations (Supplementary Table 4).

### Association between HbA1c/HDL-C ratio and cardiovascular disease by Cox regression analyses

Using the baseline 2017 HbA1c/HDL-C ratio, Kaplan-Meier cumulative hazard curves (Figure 2) demonstrated a progressive increase in CVD incidence across ascending HbA1c/HDL-C ratio quartiles. Cox regression models were fitted to further evaluate the association of HbA1c/HDL-C ratio with incident CVD. Multicollinearity among covariates in the Cox regression model was assessed using VIFs. All VIF values were below 5, confirming the absence of significant multicollinearity (Supplementary Table 5). Schoenfeld residual tests were performed for all variables included in the Cox proportional hazards regression models. Only hypertension and dyslipidemia violated the proportional hazards assumption, whereas all other variables satisfied the assumption (all *P* > 0.05) (Supplementary Table 6).

**Figure 2:**
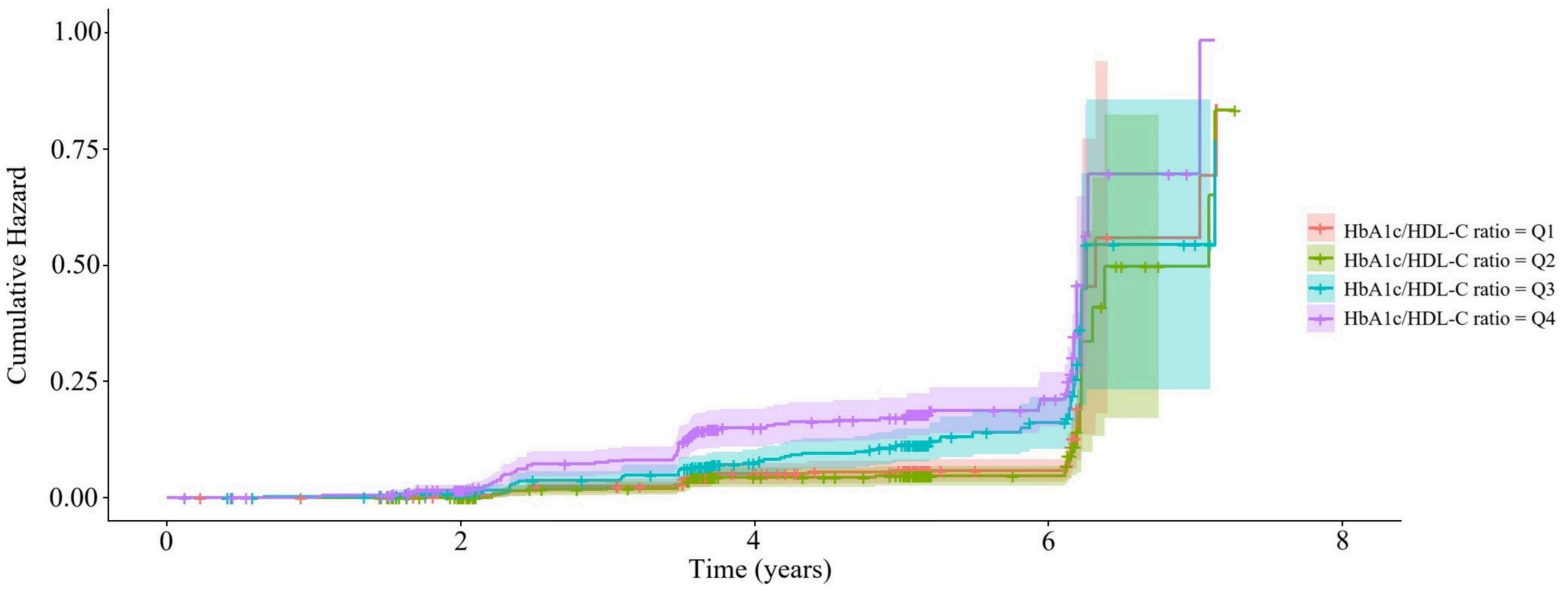
K-M plot of cardiovascular disease by HbA1c/HDL-C ratio levels. HDL-C, high-density lipoprotein cholesterol; HbA1c, hemoglobin A1c.

The association between HbA1c/HDL-C ratio and cardiovascular disease by Cox regression analyses were presented in Table 2. In the crude model (Model 1), Compared with the referent group (Q1), participants in the top quartile (Q4) of the HbA1c/HDL-C ratio was at a significantly heightened risk of CVD (HR = 2.600, 95%*CI*: 1.762–3.836, *P* < 0.001, Supplementary Table 7). The positive association endured and continued to achieve statistical significance upon progressive covariate adjustment for demographic factors (Model 2: HR = 2.076, 95%*CI*: 1.386–3.109, *P* < 0.001, Supplementary Table 8), family history of comorbidities (Model 3: HR = 2.095, 95%*CI*: 1.398–3.140, *P* = 0.011, Supplementary Table 9), and lifestyle factors (Model 4: HR = 2.680, 95%*CI*: 1.818–3.951, *P* = 0.003, Supplementary Table 10). Model 5 was adjusted for conventional demographic characteristics, family history of comorbidities, lifestyle factors, hypertension, and overweight/obesity, but excluded diabetes and dyslipidaemia, this model still demonstrated a comparable association between the highest quartile (Q4) and CVD risk (Model 5: HR = 2.029, 95%*CI*: 1.308–3.149, *P* = 0.002, Supplementary Table 11). Furthermore, additional adjustment for diabetes and dyslipidaemia on the basis of Model 5 did not attenuate this consistent association (Model 6: HR = 2.111, 95%*CI*: 1.276–3.170, *P* = 0.003, Supplementary Table 12).

**Table 2:** The association between HbA1c/HDL-C level and cardiovascular disease risk.

Variables	Q1	Q2		Q3		Q4		*P* for trend
		HR (95%*CI*)	*P*	HR (95%*CI*)	*P*	HR (95%*CI*)	*P*	
Model 1	1.000 (Reference)	0.820 (0.512–1.314)	0.410	1.602 (1.062–2.416)	0.025	2.600 (1.762–3.836)	< 0.001	0.068
Model 2	1.000 (Reference)	0.787 (0.488–1.272)	0.329	1.539 (1.009–2.348)	0.045	2.076 (1.386–3.109)	< 0.001	0.063
Model 3	1.000 (Reference)	0.792 (0.490–1.280)	0.341	1.556 (1.018–2.376)	0.041	2.095 (1.398–3.140)	< 0.001	0.053
Model 4	1.000 (Reference)	0.829 (0.502–1.414)	0.410	1.657 (1.098–2.501)	0.025	2.680 (1.818–3.951)	< 0.001	0.065
Model 5	1.000 (Reference)	0.746 (0.454–1.225)	0.247	1.409 (0.901–2.204)	0.133	2.029 (1.308–3.149)	0.002	0.080
Model 6	1.000 (Reference)	0.738 (0.449–1.214)	0.232	1.391 (0.887–2.181)	0.151	2.111 (1.276–3.170)	0.003	0.068
Model 6^a^	1.000 (Reference)	0.708 (0.433–1.157)	0.168	1.292 (0.832–2.005)	0.254	1.855 (1.194–2.880)	0.006	0.059

Model 1: crude model.

Model 2: adjusted for gender, age at screening, years, marital status, educational level, job, residential status, annual personal income.

Model 3: adjusted for gender, age at screening, years, marital status, educational level, job, residential status, annual personal income, family history of coronary heart disease, family history of hypertension, family history of diabetes.

Model 4: adjusted for gender, age at screening, years, marital status, educational level, job, residential status, annual personal income, family history of coronary heart disease, family history of hypertension, family history of diabetes, smoking status, drinking status, exercise habits, dietary taste, dietary habits, vegetable consumption frequency, fruit consumption frequency.

Model 5: adjusted for gender, age at screening, years, marital status, educational level, job, residential status, annual personal income, family history of coronary heart disease, family history of hypertension, family history of diabetes, smoking status, drinking status, exercise habits, dietary taste, dietary habits, vegetable consumption frequency, fruit consumption frequency, hypertension, overweight/obesity.

Model 6: adjusted for gender, age at screening, years, marital status, educational level, job, residential status, annual personal income, family history of coronary heart disease, family history of hypertension, family history of diabetes, smoking status, drinking status, exercise habits, dietary taste, dietary habits, vegetable consumption frequency, fruit consumption frequency, hypertension, diabetes, dyslipidemia, overweight/obesity.

Model 6^a^: Based on Model 6, only variables with *P* < 0.05 in univariate analysis were adjusted for, including age at screening, years, marital status, educational level, job, annual personal income, family history of coronary heart disease, family history of hypertension, family history of diabetes, dietary habits, vegetable consumption frequency, fruit consumption frequency, hypertension, diabetes, dyslipidemia, overweight/obesity.

HDL-C, high density lipoprotein cholesterol; HbA1c, hemoglobin A1c; HR, hazards ratio; CI, confidence interval.

To avoid overadjustment, only variables that were significantly associated with CVD risk in the univariate analyses (*P* < 0.05) were included in Model 6^a^. The association between the highest quartile (Q4) of the HbA1c/HDL-C ratio and incident CVD risk remained statistically significant, although the effect estimate was attenuated (Model 6^a^: HR = 1.855, 95%*CI*: 1.195–2.880, *P* = 0.006, Supplementary Table 13). Of note, the linear trend across quartiles was of borderline significance in all six models (*P*_for trend_ ranging from 0.053 to 0.080), with the elevated risk concentrated primarily in the highest quartile (Q4).

Given that the Kaplan–Meier cumulative hazard curves and Schoenfeld residual tests suggested potential violations of the proportional hazards assumption, stratified Cox proportional hazards regression analyses were conducted according to event time. Significant associations between the highest quartile (Q4) of the HbA1c/HDL-C ratio and incident CVD risk were observed among participants with an event time of < 3 years (HR = 4.039, 95%*CI*: 1.379–11.825, *P* = 0.011) and 3–5.99 years (HR = 3.057, 95%*CI*: 1.574–5.939, *P* < 0.001). In contrast, no statistically significant association was observed among participants with an event time of ≥6 years (Supplementary Table 14).

When HbA1c, HDL-C, and the HbA1c/HDL-C ratio were treated as continuous variables with identical covariates adjusted, HbA1c showed no significant association with CVD risk (HR = 1.094, 95%*CI*: 0.969–1.236, *P* = 0.145, Supplementary Table 15), HDL-C was inversely correlated with CVD risk (HR = 0.559, 95%*CI*: 0.380–0.822, *P* = 0.003, Supplementary Table 15), while the HbA1c/HDL-C ratio was positively correlated with CVD risk (HR = 1.291, 95%*CI*: 1.183–1.409, *P* < 0.001, Supplementary Table 15). The smaller *P*-value observed for the positive association between the HbA1c/HDL-C ratio and CVD indicates that the HbA1c/HDL-C ratio may serve as a potential biomarker for identifying CVD risk compared to HDL-C.

### Dose-response relationship between HbA1c/HDL-C ratio and cardiovascular disease by restricted cubic spline analysis

RCS analysis was performed to delineate the dose–response relationship between the HbA1c/HDL-C ratio and incident CVD. In the unadjusted model (Figure 3A), four knots were positioned at 1.99, 3.04, 3.88, and 6.11. In the adjusted model (Figure 3B), four knots were retained using the same. The pink histogram illustrates the outcome prediction incidence of study participants across HbA1c/HDL-C ratio levels. The solid light blue line represents the fitted RCS curve, and the shaded area indicates the corresponding 95%*CI*s. The red dashed line marks the estimated inflection point at an HbA1c/HDL-C ratio of 4.83 (95%*CI*: 3.55–6.06). After adjusting for confounding factors, the overall association between the HbA1c/HDL-C ratio and incident CVD was statistically significant (*P*_for overall_ < 0.001, Figure 3B), and evidence of a nonlinear association was observed (*P*_for nonlinear_ = 0.039, Figure 3B).

**Figure 3:**
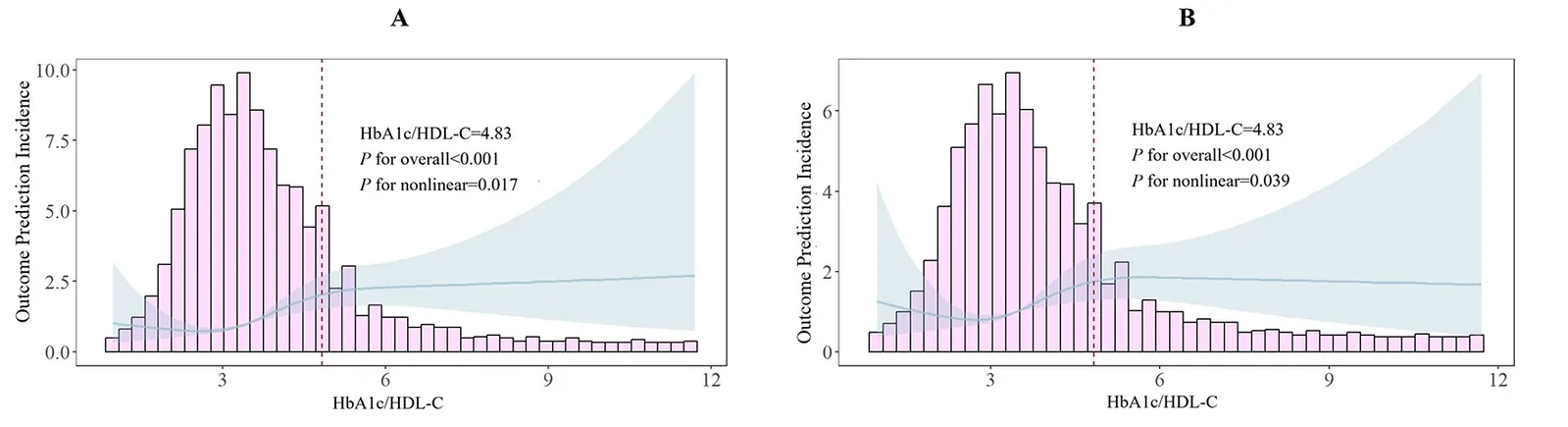
Restricted cubic spline of the association between the HbA1c/HDL-C ratio and the risk of cardiovascular disease. **(A)** crude model; **(B)** adjusted for gender, age at screening, years, marital status, educational level, job, residential status, annual personal income, family history of coronary heart disease, family history of hypertension, family history of diabetes, hypertension, diabetes, dyslipidemia, overweight/obesity, smoking status, drinking status, exercise habits, dietary taste, dietary habits, vegetable consumption frequency, fruit consumption frequency. CI, confidence interval; HR, hazard ratio; HDL-C, high-density lipoprotein cholesterol; HbA1c, hemoglobin A1c.

Stratified Cox regression analyses were subsequently performed using the estimated inflection point of 4.83 as the cutoff. A total of 1,587 participants had an HbA1c/HDL-C ratio below 4.83, with a CVD incidence rate of 9.89% (Supplementary Table 16). The remaining 288 participants exhibited a ratio above 4.83, corresponding to a CVD incidence rate of 17.36% (Supplementary Table 16). The difference in CVD incidence between the two groups was statistically significant (χ^2^ = 13.843, *P* < 0.001). Among participants with an HbA1c/HDL-C ratio < 4.83, each one-unit increase in the ratio was associated with a 44.10% higher risk of incident CVD (HR = 1.441, 95%*CI*: 1.138–1.825, *P* = 0.002). In contrast, among participants with an HbA1c/HDL-C ratio≥4.83, no significant association was observed (HR = 1.167, 95%*CI*: 0.864–1.576, *P* = 0.315). The confidence intervals widened substantially at higher HbA1c/HDL-C ratio values, consistent with the progressively smaller number of participants in this range, as reflected by the declining height of the histogram, indicating greater uncertainty in the risk estimates.

### Predictive efficacy of HbA1c/HDL-C ratio for cardiovascular disease risk

The study participants were randomly divided into test (30%) and training (70%) sets for the development and validation of Cox proportional hazards prediction models. No statistically significant differences in baseline characteristics were observed between the two sets (Supplementary Table 17). The time-dependent AUC of both models increased initially and then gradually declined, with the highest predictive performance observed at the 6-year event time (Table 3). At this time point, Model A achieved a test-set AUC of 0.854, whereas Model B achieved an AUC of 0.870. At the 7-year event time, the AUCs of both models decreased, accompanied by substantially wider 95%*CI*s. Across event time ranging from 2 to 7 years, Model B consistently achieved higher AUCs in the test set than Model A. C-statistic in the test set was also higher for Model B than for Model A (0.795 vs. 0.777), and a similar pattern was observed in the training set (0.694 vs. 0.660).

**Table 3:** Time-dependent AUC and overall C-statistic of the Cox regression-based predictive models.

Event time	AUC (95%*CI*)		C-statistic (95%*CI*)	
	Test set	Train set	Test set	Train set
Model A				
2 year	0.755 (0.642–0.868)	0.546 (0.363–0.729)	0.777 (0.737–0.817)	0.660 (0.585–0.735)
3 year	0.790 (0.736–0.844)	0.615 (0.502–0.728)		
4 year	0.830 (0.785–0.874)	0.745 (0.661–0.828)		
5 year	0.804 (0.757–0.851)	0.717 (0.631–0.802)		
6 year	0.854 (0.809–0.898)	0.719 (0.607–0.831)		
7 year	0.724 (0.652–0.876)	0.634 (0.329–0.939)		
Model B				
2 year	0.820 (0.717–0.924)	0.682 (0.504–0.861)	0.795 (0.757–0.834)	0.694 (0.626–0.763)
3 year	0.809 (0.752–0.867)	0.684 (0.582–0.787)		
4 year	0.840 (0.796–0.884)	0.767 (0.693–0.842)		
5 year	0.823 (0.778–0.867)	0.742 (0.663–0.821)		
6 year	0.870 (0.828–0.912)	0.743 (0.643–0.842)		
7 year	0.733 (0.610–0.855)	0.647 (0.346–0.948)		

Model A: The conventional model, adjusted for gender, age at screening, years, marital status, educational level, job, residential status, annual personal income, family history of coronary heart disease, family history of hypertension, family history of diabetes, hypertension, diabetes, dyslipidemia, overweight/obesity, smoking status, drinking status, exercise habits, dietary taste, dietary habits, vegetable consumption frequency, fruit consumption frequency.

Model B: The conventional model adding HbA1c/HDL-C.

AUC, area under the receiver operating characteristic curve; CI, confidence interval.

DCA demonstrated highly consistent performance of the two prediction models in the test and training sets, supporting their stability and generalizability (Supplementary Figure 2), with quantitative net benefit metrics summarized in Supplementary Table 18. For both models, the net benefit increased with longer event time duration. Net benefit was limited at the 2-year event time but increased progressively at the 3- and 4-year event time, indicating improved clinical utility over time. At the 2-year event time point, neither model demonstrated a positive net benefit, indicating that predictive risk stratification had limited clinical utility for short-term CVD event prediction. A modest positive net benefit was observed at 3 years. In the training set, the net benefit at a 30% risk threshold was 0.041 for Model A and 0.040 for Model B; however, the narrow range of applicable risk thresholds limited their practical clinical value. Both models exhibited the greatest clinical utility between 4 and 6 years, with a marked increase in net benefit over time.

Specifically, the peak net benefit of Model A in the training set increased from 0.000 at 2 years to 0.186 at 5 years, while the corresponding usable risk threshold range expanded from 0.12 to 0.69. Model B demonstrated a comparable temporal pattern, reaching a peak net benefit of 0.187 at 5 years with a usable risk threshold range of 0.68. By contrast, clinical utility declined substantially at 7 years. The peak net benefit in the training set decreased to 0.064 for Model A and 0.068 for Model B, accompanied by a marked contraction of the effective risk threshold range. Model B consistently yielded greater net benefit than Model A, with the incremental benefit of incorporating the HbA1c/HDL-C ratio becoming more apparent during mid- to long-term follow-up. Calibration plots showed acceptable agreement between predicted and observed CVD risk at the 7-year follow-up for both models, with non-significant Hosmer–Lemeshow test results (Model A: *P* = 0.312; Model B: *P* = 0.287), indicating no significant deviation between estimated and actual event probabilities.

### Comparison with multiple metabolic indexes

Table 4 presented the associations of multiple metabolic markers with incident CVD events via Cox proportional hazards regression, and their predictive performance using ROC curve analysis. Among individual metabolic parameters, FBG (HR = 1.051, 95%*CI*: 0.970–1.139, *P* = 0.224), HbA1c (HR = 1.091, 95%*CI*: 0.969–1.228, *P* = 0.151), TyG index (HR = 1.018, 95%*CI*: 0.735–1.409, *P* = 0.916) and non-HDL-C (HR = 1.011, 95%*CI*: 0.865–1.182, *P* = 0.891) showed no statistically significant association with CVD risk, while HDL-C was identified as an independent protective factor for CVD events (HR = 0.558, 95%*CI*: 0.380–0.821, *P* = 0.003). For composite metabolic indices, the HbA1c/HDL-C ratio, TG/HDL-C ratio and AIP were all significantly associated with elevated CVD risk. Notably, the HbA1c/HDL-C ratio exhibited the strongest and most robust association (HR = 1.281, 95%*CI*: 1.116–1.470, *P* < 0.001). In terms of predictive efficacy, the HbA1c/HDL-C ratio yielded the highest area under the ROC curve (AUC) of 0.636 (95%*CI*: 0.564–0.647), which was consistently higher than that of HbA1c alone (0.573), HDL-C alone (0.599), TG/HDL-C ratio (0.560), AIP (0.560) and all other individual metabolic markers evaluated in this study (Supplementary Table 19).

**Table 4:** Association analyses of various metabolic indicators with incident CVD events via Cox regression and predictive performance evaluation via ROC analysis.

Index	HR (95%*CI*)	*P*	AUC (95%*CI*)
FBG	1.051 (0.970–1.139)	0.224	0.504 (0.460–0.547)
HbA1c	1.091 (0.969–1.228)	0.151	0.573 (0.531–0.614)
HDL-C	0.558 (0.380–0.821)	0.003	0.599 (0.558–0.640)
HbA1c/HDL-C	1.281 (1.116–1.470)	< 0.001	0.636 (0.564–0.647)
TyG	1.018 (0.735–1.409)	0.916	0.482 (0.442–0.522)
TG/HDL-C	1.320 (1.087–1.602)	0.005	0.560 (0.520–0.601)
non-HDL-C	1.011 (0.865–1.182)	0.891	0.529 (0.488–0.570)
AIP	2.033 (1.024–4.035)	0.043	0.560 (0.520–0.601)

HR, hazard ratio; CI, confidence interval; AUC, area under the curve; ROC, receiver operating characteristic; FBG, fasting blood glucose; HbA1c, glycated hemoglobin A1c; HDL-C, high-density lipoprotein cholesterol; HbA1c/HDL-C, glycated hemoglobin A1c to high-density lipoprotein cholesterol ratio; TyG, triglyceride-glucose index; TG/HDL-C, triglyceride to high-density lipoprotein cholesterol ratio; non-HDL-C, non-high-density lipoprotein cholesterol; AIP, atherogenic index of plasma.

### Subgroup analysis

The associations between HbA1c/HDL-C ratio quartiles and specific CVD subtypes were presented in Table 5. Due to insufficient incident cases, Cox regression analyses were not applicable for asymptomatic coronary stenosis and valvular heart disease. For angina pectoris, participants in the highest quartile (Q4) of HbA1c/HDL-C ratio had a significantly higher incidence risk compared with those in the lowest quartile (Q1) (HR = 2.255, 95%*CI*: 1.320–3.850, *P* = 0.006), while the test for linear trend across quartiles did not reach statistical significance (*P*_for trend_ = 0.526). Similarly, the risk of myocardial infarction was markedly elevated in the Q4 group (HR = 2.830, 95%*CI*: 1.169–6.847, *P* = 0.021). For atrial fibrillation, a stepwise increase in risk was observed along with rising HbA1c/HDL-C quartiles; the highest quartile was associated with an increased risk of CVD (HR = 4.511, 95%*CI*: 1.185–17.172, *P* = 0.027), and the trend test reached statistical significance (*P*_for trend_ = 0.042). Overall, elevated HbA1c/HDL-C ratio was consistently linked to higher risk of major CVD subtypes, but all subgroup analyses presented relatively wide confidence intervals.

**Table 5:** The association between HbA1c/HDL-C ratio level and subgroup of cardiovascular disease risk.

CVD type	N (%)	Q1	Q2		Q3		Q4		*P* for trend
			*HR* (95%*CI*)	*P*	*HR* (95%*CI*)	*P*	*HR* (95%*CI*)	*P*	
Coronary heart disease									
Angina pectoris	86 (41.55)	1.000 (Reference)	0.706 (0.365–1.367)	0.302	1.253 (0.692–2.269)	0.456	2.255 (1.320–3.850)	0.006	0.526
Myocardial infarction	63 (30.43)	1.000 (Reference)	0.889 (0.327–2.421)	0.818	1.938 (0.790–4.753)	0.149	2.830 (1.169–6.847)	0.021	0.348
Asymptomatic coronary stenosis	13 (6.28)	1.000 (Reference)	—		—		—		
Atrial fibrillation	36 (17.39)	1.000 (Reference)	1.159 (0.285–4.716)	0.836	2.003 (0.558–7.184)	0.287	4.511 (1.185–17.172)	0.027	0.042
Valvular heart disease	9 (4.35)	1.000 (Reference)	—		—		—		

—, the limited number of incident cases precluded the performance of Cox regression analyses. HR, hazard ratio; CI, confidence interval.

No significant multiplicative interactions were detected (all *P*_for interaction_>0.05, Supplementary Table 20). A positive association between the HbA1c/HDL-C ratio and incident CVD persisted across the majority of subgroups. Notably, participants in the highest quartile (Q4) exhibited significantly increased CVD risk among those who were older, married, had a junior middle school education, were farmers, had an annual personal income of ≥20,000 yuan, and had no family history of coronary heart disease, hypertension, or diabetes. Significant associations were also observed among participants without hypertension, diabetes, or obesity, as well as among those without lifestyle risk factors (i.e., smoking and alcohol consumption) and those with lower vegetable intake frequency.

### Sensitivity analyses

In the Fine-Gray competing risk model accounting for mortality as a competing event, after adjustment for the same covariates as in Model 6, the sHR for the highest quartile (Q4) relative to the lowest quartile (Q1) was attenuated compared with the HR estimated from the standard Cox proportional hazards model. Nevertheless, a significant positive association between the HbA1c/HDL-C ratio and incident CVD remained (sHR = 2.007, 95%*CI*: 1.279–3.152, *P* = 0.002, Supplementary Table 21). CIF curves further validated these findings (Supplementary Figure 3).

After excluding 695 participants with HbA1c ≥6.50% and/or HDL-C < 1.0 mmol/L, 1,180 participants remained, among whom 107 (9.07%) developed CVD during follow-up. The positive association between the HbA1c/HDL-C ratio and incident CVD remained robust. Compared with participants in the lowest quartile (Q1), those in the highest quartile (Q4) had a higher risk of incident CVD (HR = 2.212, 95%*CI*: 1.162–4.214, *P* = 0.016, Supplementary Table 22), and the overall difference across quartiles was statistically significant (χ^2^ = 17.696, *P* < 0.001). RCS analyses in this restricted cohort revealed a linear relationship between HbA1c/HDL-C ratio and CVD risk (*P*_for overall_ = 0.005; *P*_for nonlinear_ = 0.167) (Supplementary Figure 4). Kaplan-Meier cumulative hazard curves showed no statistically significant association across HbA1c/HDL-C ratio quartiles (log-rank *P* = 0.078) (Supplementary Figure 5).

Furthermore, we excluded 1465 participants with hypertension, diabetes, dyslipidemia, or overweight/obesity to rule out confounding by preexisting metabolic disorders, 410 participants remained, with a CVD incidence of 5.85% (24/410). Considering that sensitivity Model 10 excluded a substantial number of participants, only Cox regression analyses were performed in this study and found that the association remained statistically significant, with the Q4 group showing a substantially elevated CVD risk (HR = 9.673, 95%*CI*: 2.620–35.722, *P* < 0.001, Supplementary Table 22). However, the confidence interval was substantially wider than that in the primary analysis because of the markedly reduced sample size and limited number of incident events, indicating reduced precision despite the persistence of a statistically significant association. Similar association were found among the excluded participants, the association between Q4 and incident CVD remained significant (HR = 4.082, 95%*CI*: 1.063–15.667, *P* = 0.040, Supplementary Table 23). The relatively limited number of incident CVD cases among the excluded participants resulted in wide confidence intervals.

## Discussion

Using data from a 7-year prospective community-based cohort in Wanzhou District, southwestern China, we investigated the association between the HbA1c/HDL-C ratio and incident CVD among older adults. We found that a higher HbA1c/HDL-C ratio was independently associated with an increased risk of CVD after adjustment for established confounders. RCS analysis demonstrated a significant nonlinear dose–response relationship, with an inflection point at a ratio of 4.83. This association remained robust across competing risk models and multiple restrictive sensitivity analyses. Furthermore, time-dependent ROC analysis, the C-statistic, and DCA collectively demonstrated that incorporating the HbA1c/HDL-C ratio into conventional risk models improved the prediction of incident CVD, highlighting its potential as a cost-effective and readily accessible biomarker for CVD risk stratification in aging populations.

The lack of effective tools for the early identification of CVD highlights the need for convenient and noninvasive serum biomarkers for risk assessment and early detection. Previous studies had provided important evidence for improving our understanding of cardiometabolic risk and identifying alternative screening approaches. Consistent with recent longitudinal findings from the China Health and Retirement Longitudinal Study (CHARLS), our results support the observation that elevated HbA1c/HDL-C ratios were correlated with an increased risk of stroke and other adverse health outcomes among older Chinese adults (12). Similarly, a nationwide prospective study in China reported similar relationship between HbA1c/HDL-C ratio levels and stroke (14). For the first time, our study extends the existing evidence by reporting a prospective association between the HbA1c/HDL-C ratio and composite cardiovascular outcomes in a rural-predominant older population after comprehensive adjustment for potential confounders.

Multivariable Cox regression analyses demonstrated an independent relationship between HbA1c/HDL-C ratio and incident CVD remained significant across progressively adjusted models. Participants in the top quartile (Q4) of the HbA1c/HDL-C ratio exhibited a markedly elevated CVD risk compared with the referent group (Q1), even after extensive adjustment for demographic, clinical, and lifestyle factors. These findings suggested that the HbA1c/HDL-C ratio may serve as an integrated marker of metabolic health, reflecting the combined effects of glycemic dysregulation and adverse lipid metabolism. Elevated HbA1c/HDL-C ratios may therefore indicate persistent metabolic and inflammatory disturbances that contribute to vascular injury and CVD development. The persistence of the association after comprehensive adjustment further indicating that HbA1c/HDL-C ratio captures residual cardiovascular risk beyond that explained by conventional risk factors.

However, after successive adjustment for hypertension, overweight/obesity, or hypertension, diabetes, dyslipidemia, and overweight/obesity, the association between the highest quartile (Q4) of the HbA1c/HDL-C ratio and incident CVD was attenuated. Model 6^a^, which included only variables that were statistically significant in the univariate analyses (*P* < 0.05), showed a further attenuation of the association.

Model 6 was a fully adjusted model constructed based on a DAG framework and included all theoretically relevant confounding variables. In contrast, Model 6^a^ included only covariates that achieved statistical significance (*P* < 0.05) in the univariate analyses. Compared with Model 6, the HRs across quartiles of the HbA1c/HDL-C ratio were generally lower in Model 6^a^, accompanied by wider confidence intervals and reduced statistical significance. These findings suggest that covariates without statistically significant univariate associations may still exert cumulative confounding effects. Reliance solely on univariate “*P*” values for covariate selection may exclude important confounding variables, thereby resulting in residual confounding and underestimation of the true association between the HbA1c/HDL-C ratio and incident CVD. The fully adjusted DAG-based model is more likely to comprehensively control for confounding bias and provide more robust estimates of the association. Furthermore, these results highlight that chronic metabolic conditions are important confounders that should not be overlooked in CVD risk assessment.

Collectively, these findings highlight the need for further mechanistic studies to elucidate the biological pathways linking the HbA1c/HDL-C ratio to CVD development. First, Prolonged hyperglycemia contributes to the production of advanced glycation end products (AGEs), which impair HDL functionality by glycating apolipoprotein A-I (apoA-I), thereby reducing cholesterol efflux capacity, paraoxonase activity, and anti-inflammatory properties (26, 27). Second, insulin resistance, which was reflected by the concomitant elevation of HbA1c and depression of HDL-C, triggered oxidative stress via NADPH oxidase activation and mitochondrial dysfunction, subsequently activating pro-inflammatory NF-κB and JNK signaling pathways that accelerate endothelial dysfunction and atherosclerosis (28). Third, low HDL-C levels in the context of hyperglycemia may indicate a shift from protective large HDL particles to dysfunctional small, dense particles with impaired reverse cholesterol transport (29). This finding implied that HbA1c/HDL-C ratio captured residual cardiovascular risk not fully reflected by conventional measures, including blood pressure, glycemic status, and lipid profiles, potentially reflecting persistent pro-atherogenic metabolic disturbances.

RCS demonstrated a significant nonlinear dose–response relationship between the HbA1c/HDL-C ratio and CVD risk, with an inflection point at 4.83. However, this threshold should be regarded as a data-driven, hypothesis-generating cut-off rather than a clinically validated threshold. Because it was derived exclusively from the distribution of the HbA1c/HDL-C ratio in our single-center cohort using spline-based modeling, external validation in large-scale, multicenter prospective studies involving diverse populations is essential before the cut-off value of 4.83 can be considered for standardized CVD risk stratification.

Despite the lack of external validation, this inflection point may still have important biological and clinical implications. Specifically, the association between the HbA1c/HDL-C ratio and incident CVD appeared to be stronger below a ratio of 4.83, whereas the risk increase tended to plateau beyond this threshold. Within this dominant population range, the composite marker of HbA1c/HDL-C ratio enables continuous, graded discrimination of CVD risk (30). The positive association observed below the inflection point suggests that the HbA1c/HDL-C ratio may serve as a sensitive indicator of metabolic risk for incident CVD across the majority of the observed exposure range. In contrast, the apparent attenuation of the association above a ratio of 4.83 should not be interpreted as evidence that cardiovascular risk no longer increases. Rather, this finding may reflect the limited number of participants and incident events at the upper end of the HbA1c/HDL-C ratio distribution, resulting in reduced statistical precision rather than a true plateau in risk. Overall, the observed nonlinear dose-response relationship supports the potential incremental prognostic value of the HbA1c/HDL-C ratio for CVD risk stratification within routine metabolic assessment, while underscoring the need for external validation in large, multicenter prospective studies before this exploratory cut-off can be translated into clinical practice.

Interestingly, our findings differed from those of the CHARLS cohort, in which a linear correlation of HbA1c-to-HDL-C with stroke risk was described (14), but are consistent with evidence of a nonlinear association between the HbA1c/HDL-C ratio and cognitive decline, with an identified inflection point of 2.06. The heterogeneity in dose–response patterns across outcomes may indicate that the metabolic pathways linking the HbA1c/HDL-C ratio to disease risk vary according to the specific clinical endpoint and population under investigation. The observed nonlinear association may partly reflect the complex dose–response relationship between HDL-C and CVD risk. At very low HDL-C levels (< 40 mg/dL), further reductions may have limited incremental effects on atherosclerotic risk because HDL particles may already be severely dysfunctional. Glycation and oxidative modification can impair the anti-inflammatory, antioxidant, and reverse cholesterol transport functions of HDL (31), thereby attenuating its vasculoprotective effects. Consequently, additional decreases in HDL-C may contribute little to further risk elevation, resulting in a plateau in the dose–response curve.

Incorporating the HbA1c/HDL-C ratio into a conventional risk model comprising established demographic, lifestyle, and clinical risk factors resulted in a modest improvement in predictive discrimination, as evidenced by increases in the C-statistic and time-dependent AUC. Decision curve analysis further demonstrated a slight increase in net clinical benefit, particularly during mid- to long-term follow-up. Although the absolute gain in discrimination was modest, this finding may still have practical implications for risk stratification in older adults. Traditional risk prediction models often exhibit reduced discriminatory performance in this population, whereas the HbA1c/HDL-C ratio can be readily derived from routinely measured laboratory parameters without incurring additional cost.

Our findings suggest that the HbA1c/HDL-C ratio, an integrated glucolipid biomarker, may serve as a useful supplementary metabolic indicator for CVD risk assessment in older adult populations. By integrating information from both glucose and lipid metabolism, the HbA1c/HDL-C ratio may better reflect the overall metabolic disturbances underlying atherosclerosis than individual glycemic markers such as FBG and HbA1c, which capture only glycemic status (32) and exhibited limited predictive performance in our cohort. However„ the TyG index, a widely used surrogate marker of insulin resistance (33) was not significantly associated with incident CVD in our study, possibly because of the specific characteristics of the study population and the limited number of CVD events.

The subgroup analyses provide additional support for the association between the HbA1c/HDL-C ratio and incident CVD while underscoring the exploratory nature of these findings. In analyses of individual CVD subtypes, a higher HbA1c/HDL-C ratio was consistently associated with increased risks of angina pectoris, myocardial infarction, and atrial fibrillation, suggesting that this integrated glucolipid biomarker may reflect multiple pathophysiological processes involved in CVD development (15), including atherosclerotic coronary disease, myocardial ischemia, and atrial remodeling. Notably, atrial fibrillation exhibited a significant dose-response relationship across HbA1c/HDL-C ratio quartiles, suggesting that combined disturbances in glucose and lipid metabolism may be particularly relevant to the development of atrial arrhythmias (34). Nevertheless, these subtype-specific findings should be interpreted cautiously because of the limited number of events and require confirmation in larger prospective studies.

The subgroup analyses across different variables demonstrated that the association between the HbA1c/HDL-C ratio and incident CVD risk remained significant among participants without major cardiometabolic risk factors, including hypertension, diabetes, and overweight/obesity, as well as among those with favorable lifestyle characteristics, such as non-smoking and non-drinking. Notably, these individuals were generally considered to have a relatively low baseline cardiovascular risk, suggesting that the HbA1c/HDL-C ratio may identify residual metabolic vulnerability that is not captured by conventional cardiovascular risk indicators.

Sensitivity analyses further supported the robustness of the primary findings. Excluding participants with marked glycemic or lipid abnormalities did not materially alter the observed association. Moreover, the association remained significant after excluding individuals with established metabolic disorders, suggesting that the observed increase in CVD risk was not driven by extreme metabolic abnormalities. Importantly, the Fine–Gray competing risk analysis yielded results consistent with those of the conventional Cox proportional hazards model, with higher HbA1c/HDL-C ratios remaining significantly associated with an increased risk of incident CVD. Collectively, these findings support the robustness of the observed association and suggest that the HbA1c/HDL-C ratio may serve as an early indicator of CVD risk, even before the development of overt metabolic disorders.

Nevertheless, the current results should be interpreted with caution. First, the number of incident cases for each CVD subtype was relatively small, resulting in wide confidence intervals and limited statistical power for most trend tests; thus the magnitude of risk for specific CVD endpoints cannot be precisely estimated. Second, the second sensitivity model, which excluded participants with common metabolic conditions, retained only 410 participants and 24 CVD events, leading to an extremely wide confidence interval and a large point estimate of hazard ratio. Therefore, the significant associations observed in subtype analyses and the restrictive sensitivity model should be regarded as preliminary evidence rather than definitive conclusions. Large-scale, multicenter prospective cohort studies with sufficient endpoint events are required to verify the stability, magnitude and generalizability of these findings.

An important advantage of the HbA1c/HDL-C ratio is its feasibility for widespread clinical implementation. Because both HbA1c and HDL-C are routinely measured during annual health examinations under China's National Basic Public Health Service Program, the ratio can be obtained without additional testing, equipment, or healthcare expenditure. Moreover, HbA1c assessment does not require fasting, enhancing its practicality in older populations. The standardized measurement of both biomarkers across clinical laboratories further supports the reliability and reproducibility of the ratio. Compared with novel biomarkers such as hs-CRP and Lp(a), which are not universally available in primary care settings, the HbA1c/HDL-C ratio can be derived from routinely collected laboratory data, facilitating its integration into existing cardiovascular risk assessment programs. Given its low cost, accessibility, and scalability, this biomarker may represent a valuable tool for population-based cardiovascular risk stratification, particularly in resource-limited settings.

The public health implications of these findings are particularly relevant in the context of population aging and growing cardiovascular disease burden. In rural areas of western China, where access to advanced diagnostic resources may be limited, the HbA1c/HDL-C ratio represents a low-cost and readily available biomarker that could be integrated into current community-based screening initiatives. The exploratory threshold of 4.83 may serve as a reference for identifying individuals at elevated metabolic risk in epidemiological research; however, it is not intended to guide clinical decision-making. Whether targeted monitoring and lifestyle interventions among individuals with elevated HbA1c/HDL-C ratios can reduce the incidence of CVD remains to be established in prospective interventional studies. However, given its accessibility, scalability, and minimal implementation burden, the HbA1c/HDL-C ratio may represent as a valuable supplementary marker to current cardiovascular risk assessment strategies, particularly in resource-constrained primary healthcare settings.

Consistent with the findings from the time-dependent ROC analysis, the C-statistic, and decision curve analysis (DCA), our study showed that incorporating the HbA1c/HDL-C ratio into a conventional risk model comprising established demographic, lifestyle, and clinical cardiovascular risk factors improved the prediction of incident CVD. The model demonstrated stable discrimination and clinical utility in both the training and test datasets, with no evidence of substantial overfitting, supporting the robustness and internal validity of the findings. These results should be interpreted in the context of the inherent challenges associated with cardiovascular risk prediction in older adults. In this population, age exerts a dominant influence on absolute risk, often attenuating the discriminatory capacity of traditional risk factors and resulting in C-statistics that commonly range between 0.60 and 0.65 (35). Therefore, even modest improvements in discrimination may have practical value, particularly when derived from inexpensive and routinely available biomarkers (36).

Several avenues for future research warrant consideration. First, multicenter prospective studies involving geographically and ethnically diverse Chinese populations are needed to validate the generalizability of our findings. Second, the enhanced prognostic performance attributable to HbA1c-to-HDL-C incorporation should be evaluated in combination with emerging biomarkers of inflammation and residual cardiovascular risk, including hs-CRP and Lp(a). Third, mechanistic studies are required to elucidate the biological pathways linking an elevated HbA1c/HDL-C ratio to atherosclerosis and CVD development. Finally, randomized controlled trials and implementation studies should determine whether interventions targeting glucolipid metabolic dysfunction can modify the HbA1c/HDL-C ratio and, ultimately, reduce CVD risk in older adults.

Several strengths of this study merit consideration. First, the prospective cohort design, extended 7-year follow-up, and rigorous outcome ascertainment based on both participant-reported physician diagnoses and linkage to a regional surveillance system substantially enhanced the reliability of outcome classification. Furthermore, this study provides prospective evidence from a rural-predominant older population in southwestern China. Although we do not propose the HbA1c/HDL-C ratio as a novel biomarker, our findings extend previous evidence from the CHARLS and other cohorts by demonstrating its longitudinal association with composite CVD outcomes in this distinct demographic and geographic population. Moreover, unlike studies based on publicly available databases, our findings were derived from original field-collected data obtained through standardized measurements and rigorous quality control procedures, thereby enhancing the regional relevance and population specificity of the evidence. Second, the robustness of the observed associations was supported by progressively adjusted Cox regression models and formal verification of the proportional hazards assumption. Third, the availability of detailed information on lifestyle behaviors, socioeconomic characteristics, and clinical factors allowed comprehensive adjustment for potential confounders, strengthening the credibility of the findings.

Several limitations should be considered when interpreting the findings of this study. First, because the cohort was recruited from a single district in southwestern China, and focused on olderly participants as the study population, the generalizability of the results to other geographic regions and ethnic populations may be limited. Nevertheless, the predominantly rural and aging characteristics of the study population reflect the demographic profile of many communities in western China.

Second, although extensive adjustment was performed for demographic, socioeconomic, lifestyle, and clinical factors, residual confounding from unmeasured variables, including genetic factors, psychosocial determinants, and environmental exposures, remains possible.

Third, outcome ascertainment partly relied on self-reported physician diagnoses, which may have introduced information bias despite validation through linkage with the regional surveillance system.

Fourth, owing to the limited number of events, analyses of individual CVD subtypes were not feasible, restricting our ability to evaluate potential heterogeneity across specific cardiovascular outcomes.

Fifth, the primary composite endpoint comprised heterogeneous cardiovascular conditions with potentially distinct underlying biological mechanisms. Although the validated events were predominantly acute coronary events (i.e., angina pectoris, myocardial infarction and atrial fibrillation), based on the available ICD-10 validation codes, the broader clinical endpoint also encompassed valvular heart disease. Future studies should, where event numbers permit, conduct exploratory analyses of atherosclerotic CVD outcomes (e.g., cardiovascular death) as separate endpoints.

Sixth, Model 6 included 22 predictor variables and 207 incident CVD events, yielding an events-per-variable (EPV) ratio of approximately 9.4. Although this value is slightly below the commonly recommended threshold of 10–15 events per variable, all VIF values were below 5, indicating the absence of substantial multicollinearity. The robustness of the primary findings was further supported by Model 6^a^, which included only variables that were statistically significant in the univariate Cox proportional hazards regression analyses (*P* < 0.05) and yielded consistent results despite reduced precision. Nevertheless, future studies with larger numbers of incident events are warranted to validate these findings in fully adjusted models.

Seventh, 1,596 of the 3,471 initially screened participants were excluded from the final analysis. Comparisons of baseline characteristics revealed statistically significant differences between included and excluded participants for most variables. This relatively high exclusion rate may have introduced selection bias and potentially limited the generalizability of the findings to healthier or more compliant older adults. However, the primary reason for exclusion was age < 60 years, reflecting the study's focus on older adults rather than systematic health-related selection. Moreover, only 37 incident CVD cases were observed among the excluded participants, corresponding to a substantially lower incidence rate than that observed in the older study population. Supplementary analyses conducted in the excluded subgroup further supported the robustness of the observed association between the HbA1c/HDL-C ratio and incident CVD.

Finally, multiple subgroup and sensitivity analyses were performed, which may have increased the risk of Type I error inflation. However, these analyses were pre-specified and hypothesis-driven rather than exploratory data mining. We did not apply formal multiplicity adjustments (e.g., Bonferroni correction) to maintain statistical power, but we caution that findings from subgroup analyses should be interpreted as exploratory and require confirmation in independent cohorts. Our study demonstrated a significant association between the HbA1c/HDL-C ratio and incident CVD. However, the generalizability and clinical applicability of these findings should be interpreted with caution. Given the limited body of available evidence, the HbA1c/HDL-C ratio should not be overinterpreted as an established cardiovascular risk marker. At present, it is best considered a complementary indicator that may assist, rather than replace, conventional cardiovascular risk assessment approaches.

## Conclusion

In this prospective cohort study with 7 years of follow-up, elevated HbA1c/HDL-C ratio was independently linked to an increased risk of incident CVD among community-dwelling older adults in Wanzhou District, China. A significant nonlinear dose–response relationship was observed, with an apparent risk threshold at an HbA1c/HDL-C ratio of 4.83. Furthermore, the addition of the HbA1c/HDL-C ratio to conventional risk markers yielded modest improvements in risk discrimination and reclassification. Given its accessibility, low cost, and reliance on routinely measured laboratory parameters, HbA1c/HDL-C ratio may serve as a cost-effective supplementary tool for community-level CVD risk stratification, particularly in resource-constrained primary healthcare environments. Further research is warranted to confirm its utility among metabolically healthy older adults.

## Data Availability

The original contributions presented in the study are included in the article/Supplementary material, further inquiries can be directed to the corresponding author.
