# Supplementary material for: HbA1c-to-HDL-C ratio and risk of incident cardiovascular disease among older adults: evidence from a 7-year prospective cohort study in southwestern China
Source: Front Public Health. 2026 Sep 17;14:1901063. doi: 10.3389/fpubh.2026.1901063 (PMC13626905; doi:10.3389/fpubh.2026.1901063)
Supplement: Supplementary file 1 [file Data_Sheet_2.pdf]

Supplementary Tables of Contents

Title	Page
Supplementary Table 1 Comparison of baseline characteristics between included and excluded participants	3
Supplementary Table 2 The total CVD events integrated by the two approaches.	5
Supplementary Table 3 Baseline characteristics according to the quartiles of the HbA1c/HDL-C ratio.	6
Supplementary Table 4 Measured values of different indicators in participants with or without cardiovascular disease.	9
Supplementary Table 5 Assessment of multicollinearity using VIF.	10
Supplementary Table 6 Assessment of the proportional hazards assumption by Schoenfeld residual tests.	11
Supplementary Table 7 Association between HbA1c/HDL-C ratio and cardiovascular disease risk by cox regression analyses of Model 1.	12
Supplementary Table 8 Association between HbA1c/HDL-C ratio and cardiovascular disease risk by cox regression analyses of Model 2.	13
Supplementary Table 9 Association between HbA1c/HDL-C ratio and cardiovascular disease risk by cox regression analyses of Model 3.	14
Supplementary Table 10 Association between HbA1c/HDL-C ratio and cardiovascular disease risk by cox regression analyses of Model 4.	16
Supplementary Table 11 Association between HbA1c/HDL-C ratio and cardiovascular disease risk by cox regression analyses of Model 5.	18

Supplementary Table 12 Association between HbA1c/HDL-C ratio and cardiovascular disease risk by cox regression analyses of Model 6.	20
Supplementary Table 13 Association between HbA1c/HDL-C ratio and cardiovascular disease risk by cox regression analyses of Model 6[a].	22
Supplementary Table 14 Association between HbA1c/HDL-C ratio and cardiovascular disease risk by cox regression analyses stratified by event time.	24
Supplementary Table 15 The association between HbA1c, HDL-C or HbA1c/HDL-C level and the risk of cardiovascular disease.	25
Supplementary Table 16 Subgroup analyses for the association between the HbA1c/HDL-C ratio and incident CVD stratified by the inflection point identified via restricted cubic spline analysis.	29
Supplementary Table 17 Baseline characteristic comparison between the training set and test set grouped for the Cox regression prediction model.	30
Supplementary Table 18 Quantitative net benefit and net drift values of time-dependent DCA for Model A and Model B.	32
Supplementary Table 19 ROC analyses of different metabolic indexes for incident CVD events.	33
Supplementary Table 20 Subgroup and interaction analyses of HbA1c/HDL-C ratio and cardiovascular disease risk.	34
Supplementary Table 21 The HbA1c/HDL-C and mortality in competing risk analyses.	37
Supplementary Table 22 Sensitivity analyses for the association between HbA1c/HDL-C level and cardiovascular disease risk.	38
Supplementary Table 23 Association between HbA1c/HDL-C ratio and cardiovascular disease risk by cox regression analyses among excluded participants.	39

Supplementary Table 1 Comparison of baseline characteristics between included and excluded participants.

Variables	Total (n=3471)	Inclusion (n=1875)	Exclusion (n=1596)	Statistic	*P*
Gender				χ^2=13.513	<0.001
Males	1790 (51.57)	913 (51.01)	877 (48.99)		
Females	1681 (48.43)	962 (57.23)	719 (42.77)		
Age at screening, years	65.00 (54.00, 73.00)	72.00 (67.00, 76.00)	53.00 (50.00, 58.00)	*Z*=-5.226	<0.001
Marital status				χ^2=118.325	<0.001
Separated/divorced/widowed/unmarried	323 (9.46)	270 (83.59)	53 (16.41)		
Married/cohabiting	3090 (90.54)	1605 (51.94)	1485 (48.06)		
Educational level				χ^2=634.143	<0.001
Primary school and below	1477 (43.28)	1163 (78.74)	314 (21.26)		
Junior middle school	1180 (34.57)	499 (42.29)	681 (57.71)		
Technical secondary/high school	555 (16.26)	150 (27.03)	405 (72.97)		
College and above	201 (5.89)	63 (31.34)	138 (68.66)		
Job				χ^2=101.309	<0.001
Others	627 (18.06)	225 (35.89)	402 (64.11)		
Farmers	2844 (81.94)	1650 (58.02)	1194 (41.98)		
Residential status				χ^2=409.450	<0.001
Living alone	279 (8.17)	215 (77.06)	64 (22.94)		
Living only with spouse	1603 (46.97)	1074 (67.00)	529 (33.00)		
Living only with children	154 (4.51)	114 (74.03)	40 (25.97)		
Living with spouse and children	1377 (40.35)	472 (34.28)	905 (65.72)		
Annual personal income, yuan				χ^2=698.513	<0.001
＜4,999	895 (26.22)	723 (80.78)	172 (19.22)		
5,000-9,999	556 (16.29)	421 (75.72)	135 (24.28)		
10,000-19,999	321 (9.41)	206 (64.17)	115 (35.83)		
≥20 000	1641 (48.08)	525 (31.99)	1116 (68.01)		
Family history of coronary heart disease				χ^2=1.683	0.195
No	3196 (93.59)	1764 (55.19)	1432 (44.81)		
Yes	219 (6.41)	111 (50.68)	108 (49.32)		
Family history of hypertension				χ^2=33.296	<0.001
No	2654 (77.72)	1527 (57.54)	1127 (42.46)		
Yes	761 (22.28)	348 (45.73)	413 (54.27)		
Family history of diabetes				χ^2=9.026	0.003
No	3166 (92.71)	1761 (55.62)	1405 (44.38)		
Yes	249 (7.29)	114 (45.78)	135 (54.22)		
Hypertension				χ^2=200.079	<0.001
No	1870 (54.76)	822 (43.96)	1048 (56.04)		
Yes	1545 (45.24)	1053 (68.16)	492 (31.84)		
Diabetes				χ^2=25.266	<0.001
No	3036 (88.90)	1621 (53.39)	1415 (46.61)		

Yes	379 (11.10)	254 (67.02)	125 (32.98)		
Dyslipidemia				χ^2=0.019	0.890
No	2399 (70.25)	1319 (54.98)	1080 (45.02)		
Yes	1016 (29.75)	556 (54.72)	460 (45.28)		
Overweight/obesity				χ^2=60.962	<0.001
No	1870 (54.77)	1140 (60.96)	730 (39.04)		
Yes	1544 (45.23)	735 (47.60)	809 (52.40)		
Smoking status				χ^2=2.148	0.143
No	2860 (83.75)	1586 (55.45)	1274 (44.55)		
Yes	555 (16.25)	289 (52.07)	266 (47.93)		
Drinking status				χ^2=63.380	<0.001
Non-drinker	2834 (82.99)	1641 (57.90)	1193 (42.10)		
Light drinking	435 (12.74)	184 (42.30)	251 (57.70)		
Heavy drinking regularly	146 (4.28)	50 (34.25)	96 (65.75)		
Exercise habits				χ^2=40.495	<0.001
Regular exercise	2880 (84.33)	1514 (52.57)	1366 (47.43)		
Insufficient exercise	535 (15.67)	361 (67.48)	174 (32.52)		
Dietary taste				χ^2=37.030	<0.001
Moderate	2806 (82.17)	1473 (52.49)	1333 (47.51)		
Light taste	418 (12.24)	274 (65.55)	144 (34.45)		
Salty taste	191 (5.59)	128 (67.02)	63 (32.98)		
Dietary habits				χ^2=46.504	<0.001
Balanced diet	2975 (87.12)	1569 (52.74)	1406 (47.26)		
Vegetarian-biased diet	406 (11.89)	287 (70.69)	119 (29.31)		
Meat-biased diet	34 (1.00)	19 (55.88)	15 (44.12)		
Vegetable consumption frequency				χ^2=65.247	<0.001
≤2 days per week	2571 (75.29)	1413 (54.96)	1158 (45.04)		
3-4 days per week	663 (19.41)	315 (47.51)	348 (52.49)		
≥5 days per week	181 (5.30)	147 (81.22)	34 (18.78)		
Fruit consumption frequency				χ^2=144.941	<0.001
≤2 days per week	1087 (31.83)	496 (45.63)	591 (54.37)		
3-4 days per week	624 (18.27)	269 (43.11)	355 (56.89)		
≥5 days per week	1704 (49.90)	1110 (65.14)	594 (34.86)		
HbA1c/HDL-C ratio, M (Q_1, Q_3)	3.54 (2.82, 4.45)	3.46 (2.78, 4.33)	3.71 (2.87, 4.59)	Z=-4.258	<0.001
New CVD				χ^2=100.35	<0.001
No	3227 (92.97)	1668 (51.69)	1559 (48.31)		
Yes	244 (7.03)	207 (84.84)	37 (15.16)		

Z: Mann-Whitney test, χ^2: Chi-square test

Abbreviations: M: Median; Q1. Quartile 1; Q2. Quartile 2; Q3. Quartile 3; Q4. Quartile 4; HDL-C. high density lipoprotein cholesterol; HbA1c. hemoglobin A1c; CVD. cardiovascular disease.

Supplementary Table 2 The total CVD events integrated by the two approaches.

CVD type	ICD10	Case	%
Coronary heart disease			
Angina pectoris	I20	86	41.55
Myocardial infarction	I21–I22	63	30.43
Asymptomatic coronary stenosis	I24	13	6.28
Atrial fibrillation	I48	36	17.39
Valvular heart disease	I05–I09, I34–I39	9	4.35
Total		207	

Abbreviations: CVD.cardiovascular diseases; ICD-10international classification of diseases-10 edition

Supplementary Table 3 Baseline characteristics according to the quartiles of the HbA1c/HDL-C ratio.

Variables	Total (n=1,875)	Q1 (n=469)	Q2 (n=473)	Q3 (n=469)	Q4 (n=464)	Statistic	*P*
Gender						12.035	0.007
Males	913 (48.69)	201 (22.02)	223 (24.42)	247 (27.05)	242 (26.51)		
Females	962 (51.31)	268 (27.86)	250 (25.99)	222 (23.08)	222 (23.08)		
Age at screening, years						10.766	0.292
60-64	282 (15.04)	63 (22.34)	65 (23.05)	70 (24.82)	84 (29.79)		
65-69	419 (22.35)	104 (24.82)	110 (26.25)	101 (24.11)	104 (24.82)		
70-74	531 (28.32)	127 (23.92)	125 (23.54)	145 (27.31)	134 (25.24)		
≥75	643 (34.29)	175 (27.22)	173 (26.91)	153 (23.79)	142 (22.08)		
Marital status						2.628	0.453
Separated/divorced/widowed/unmarried	270 (14.40)	70 (25.93)	77 (28.52)	63 (23.33)	60 (22.22)		
Married/cohabiting	1605 (85.60)	399 (24.86)	396 (24.67)	406 (25.30)	404 (25.17)		
Educational level						36.523	<0.001
Primary school and below	1163 (62.03)	328 (28.20)	306 (26.31)	268 (23.04)	261 (22.44)		
Junior middle school	499 (26.61)	107 (21.44)	127 (25.45)	138 (27.66)	127 (25.45)		
Technical secondary/high school	150 (8.00)	25 (16.67)	29 (19.33)	43 (28.67)	53 (35.33)		
College and above	63 (3.36)	9 (14.29)	11 (17.46)	20 (31.75)	23 (36.51)		
Job						51.332	<0.001
Others	225 (12.00)	26 (11.56)	42 (18.67)	65 (28.89)	92 (40.89)		
Farmers	1650 (88.00)	443 (26.85)	431 (26.12)	404 (24.48)	372 (22.55)		
Residential status						8.451	0.489
Living alone	215 (11.47)	54 (25.12)	63 (29.30)	48 (22.33)	50 (23.26)		
Living only with spouse	1074 (57.28)	277 (25.79)	263 (24.49)	270 (25.14)	264 (24.58)		
Living only with children	114 (6.08)	36 (31.58)	25 (21.93)	27 (23.68)	26 (22.81)		
Living with spouse and children	472 (25.17)	102 (21.61)	122 (25.85)	124 (26.27)	124 (26.27)		

Annual personal income, yuan						75.919	<0.001
＜4,999	723 (38.56)	201 (27.80)	200 (27.66)	189 (26.14)	133 (18.40)		
5,000-9,999	421 (22.45)	134 (31.83)	105 (24.94)	84 (19.95)	98 (23.28)		
10,000-19,999	206 (10.99)	46 (22.33)	62 (30.10)	50 (24.27)	48 (23.30)		
≥20 000	525 (28.00)	88 (16.76)	106 (20.19)	146 (27.81)	185 (35.24)		
Family history of coronary heart disease						10.143	0.017
No	1764 (94.08)	450 (25.51)	452 (25.62)	436 (24.72)	426 (24.15)		
Yes	111 (5.92)	19 (17.12)	21 (18.92)	33 (29.73)	38 (34.23)		
Family history of hypertension						15.299	0.002
No	1527 (81.44)	399 (26.13)	401 (26.26)	367 (24.03)	360 (23.58)		
Yes	348 (18.56)	70 (20.11)	72 (20.69)	102 (29.31)	104 (29.89)		
Family history of diabetes						17.401	<0.001
No	1761 (93.92)	453 (25.72)	451 (25.61)	437 (24.82)	420 (23.85)		
Yes	114 (6.08)	16 (14.04)	22 (19.30)	32 (28.07)	44 (38.60)		
Hypertension						34.069	<0.001
No	822 (43.84)	251 (30.54)	218 (26.52)	187 (22.75)	166 (20.19)		
Yes	1053 (56.16)	218 (20.70)	255 (24.22)	282 (26.78)	298 (28.30)		
Diabetes						147.666	<0.001
No	1621 (86.45)	451 (27.82)	434 (26.77)	408 (25.17)	328 (20.23)		
Yes	254 (13.55)	18 (7.09)	39 (15.35)	61 (24.02)	136 (53.54)		
Dyslipidemia						82.674	<0.001
No	1319 (70.35)	351 (26.61)	371 (28.13)	347 (26.31)	250 (18.95)		
Yes	556 (29.65)	118 (21.22)	102 (18.35)	122 (21.94)	214 (38.49)		
Overweight/obesity						98.341	<0.001
No	1140 (60.80)	345 (30.26)	312 (27.37)	283 (24.82)	200 (17.54)		
Yes	735 (39.20)	124 (16.87)	161 (21.90)	186 (25.31)	264 (35.92)		

Smoking status						8.546	0.036
No	1586 (84.59)	413 (26.04)	405 (25.54)	388 (24.46)	380 (23.96)		
Yes	289 (15.41)	56 (19.38)	68 (23.53)	81 (28.03)	84 (29.07)		
Drinking status						4.232	0.645
Non-drinker	1641 (87.52)	416 (25.35)	400 (24.38)	411 (25.05)	414 (25.23)		
Light drinking	184 (9.81)	39 (21.20)	50 (27.17)	50 (27.17)	45 (24.46)		
Heavy drinking regularly	50 (2.67)	14 (28.00)	14 (28.00)	8 (16.00)	14 (28.00)		
Exercise habits						1.397	0.706
Regular exercise	1514 (80.75)	371 (24.50)	388 (25.63)	378 (24.97)	377 (24.90)		
Insufficient exercise	361 (19.25)	98 (27.15)	85 (23.55)	91 (25.21)	87 (24.10)		
Dietary taste						9.498	0.147
Moderate	1473 (78.56)	369 (25.05)	383 (26.00)	362 (24.58)	359 (24.37)		
Light taste	274 (14.61)	74 (27.01)	56 (20.44)	70 (25.55)	74 (27.01)		
Salty taste	128 (6.83)	26 (20.31)	25 (19.53)	37 (28.91)	40 (31.25)		
Dietary habits						-	0.285*
Balanced diet	1569 (83.68)	391 (24.92)	404 (25.75)	384 (24.47)	390 (24.86)		
Vegetarian-biased diet	287 (15.31)	75 (26.13)	55 (19.16)	79 (27.53)	78 (27.18)		
Meat-biased diet	19 (1.01)	3 (15.79)	5 (26.32)	6 (31.58)	5 (26.32)		
Vegetable consumption frequency						12.096	0.060
≤2 days per week	1413 (75.36)	369 (26.11)	346 (24.49)	348 (24.63)	350 (24.77)		
3-4 days per week	315 (16.80)	73 (23.17)	68 (21.59)	86 (27.30)	88 (27.94)		
≥5 days per week	147 (7.84)	27 (18.37)	50 (34.01)	35 (23.81)	35 (23.81)		
Fruit consumption frequency						94.026	<0.001
≤2 days per week	496 (26.45)	70 (14.11)	180 (36.29)	146 (29.44)	100 (20.16)		
3-4 days per week	269 (14.35)	57 (21.19)	56 (20.82)	80 (29.74)	76 (28.25)		
≥5 days per week	1110 (59.20)	342 (30.81)	228 (20.54)	243 (21.89)	297 (26.76)		

Abbreviations: Q1. Quartile 1; Q2. Quartile 2; Q3. Quartile 3; Q4. Quartile 4; *Fisher exact test

Supplementary Table 4 Measured values of different indicators in participants with or without cardiovascular disease.

Variables	Total	Non-CVD	CVD	Statistic	*P*
Body mass index, kg/m^2	23.14 (21.09, 25.33)	23.01 (21.01, 25.25)	23.92 (22.05, 25.69)	Z=-3.653	<0.001
Mean systolic blood pressure, mmHg	135.00 (125.50, 149.00)	135.00 (126.00, 148.50)	135.50 (124.00, 154.25)	Z=-0.502	0.616
Mean diastolic blood pressure, mmHg	79.00 (73.00, 85.00)	79.00 (72.50, 85.00)	79.00 (73.50, 84.75)	Z=-0.622	0.534
Fasting blood glucose, mmol/L	5.00 (4.60, 5.50)	5.00 (4.60, 5.50)	5.00 (4.60, 5.60)	Z=-0.171	0.865
Glycated hemoglobin, %	5.40 (5.00, 5.90)	5.40 (5.00, 5.90)	5.70 (5.10, 6.30)	Z=-3.469	<0.001
Triglyceride, mmol/L	1.31 (0.97, 1.89)	1.31 (0.97, 1.89)	1.29 (1.00, 1.86)	Z=-0.593	0.553
Total cholesterol, mmol/L	4.69 (4.12, 5.37)	4.52 (4.16, 5.40)	4.71 (3.79, 5.19)	Z=-3.363	<0.001
Low-density lipoprotein cholesterol, mmol/L	2.33 (1.77, 2.97)	2.24 (1.79, 2.99)	2.34 (1.59, 2.83)	Z=-2.403	0.016
High-density lipoprotein cholesterol, mmol/L	1.58 (1.33, 1.90)	1.60 (1.35, 1.92)	1.44 (1.23, 1.72)	Z=-4.748	<0.001
HbA1c/HDL-C ratio level	3.45 (2.76, 4.32)	3.40 (2.74, 4.24)	3.93 (3.06, 4.79)	Z=-5.066	<0.001

Abbreviations: CVD. cardiovascular disease; HDL-C. high density lipoprotein; HbA1c. hemoglobin A1c.

Supplementary Table 5 Assessment of multicollinearity using VIF.

Variable	Tolerance	VIF
Gender	0.730	1.369
Age at screening, years	0.848	1.179
Marital status	0.873	1.146
Educational level	0.655	1.528
Job	0.673	1.487
Residential status	0.845	1.184
Annual personal income, yuan	0.490	2.039
Family history of coronary heart disease	0.838	1.194
Family history of hypertension	0.727	1.375
Family history of diabetes	0.831	1.204
Hypertension	0.879	1.137
Diabetes	0.865	1.156
Dyslipidemia	0.896	1.116
Overweight/obesity	0.907	1.102
Smoking status	0.668	1.498
Drinking status	0.747	1.338
Exercise habits	0.860	1.163
Dietary taste	0.424	2.359
Dietary habits	0.431	2.322
Fruit consumption frequency	0.863	1.159
Vegetable consumption frequency	0.530	1.888
HbA1c/HDL-C ratio level	0.818	1.223

Abbreviations: VIF. variance inflation factor

Supplementary Table 6 Assessment of the proportional hazards assumption by Schoenfeld residual tests.

Variables	χ^2	*P*
Gender	1.361	0.243
Age at screening	2.010	0.156
Marital status	0.267	0.605
Educational level	2.434	0.119
Job	0.255	0.236
Residential status	0.042	0.837
Annual personal income	0.522	0.470
Hypertension	5.016	0.025
Dyslipidemia	7.375	0.007
Diabetes	3.789	0.052
Smoking status	0.358	0.550
Drinking status	1.013	0.314
Exercise habits	0.082	0.774
Family history of coronary heart disease	0.100	0.752
Family history of hypertension	0.079	0.778
Family history of diabetes	0.440	0.269
Overweight/obesity	2.877	0.090
HbA1c/HDL.C ration	0.806	0.369
Dietary taste	2.942	0.086
Dietary habits	0.648	0.335
Vegetable consumption frequency	0.004	0.949
Fruit consumption frequency	2.017	0.156
GLOBAL	4.032	0.048

Supplementary Table 7 Association between HbA1c/HDL-C ratio and cardiovascular disease risk by cox regression analyses of Model 1.

Variables	*β*	*S.E*	*Z*	*P*	HR (95%*CI*)
HbA1c/HDL-C ratio level					
Q1					1.000 (Reference)
Q2	-0.198	0.240	-0.823	0.410	0.820 (0.512-1.314)
Q3	0.471	0.210	2.247	0.025	1.602 (1.062-2.416)
Q4	0.955	0.198	4.815	<0.001	2.600 (1.762-3.836)

Abbreviations: Q1. Quartile 1; Q2. Quartile 2; Q3. Quartile 3; Q4. Quartile 4; HDL-C. high density lipoprotein cholesterol; HbA1c. hemoglobin A1c; HR: Hazards Ratio, CI: Confidence Interval; S.E. standard error;

Model 1:unadjusted.

Supplementary Table 8 Association between HbA1c/HDL-C ratio and cardiovascular disease risk by cox regression analyses of Model 2.

Variables	*β*	*S.E*	*Z*	*P*	HR (95%*CI*)
HbA1c/HDL-C ratio level					
Q1					1.000 (Reference)
Q2	-0.239	0.245	-0.977	0.329	0.787 (0.488-1.272)
Q3	0.431	0.215	2.002	0.045	1.539 (1.009-2.348)
Q4	0.730	0.206	3.543	<0.001	2.076 (1.386-3.109)
Gender					
Males					1.000 (Reference)
Females	0.159	0.148	1.078	0.281	1.173 (0.878-1.566)
Age at screening, years					
60-64					1.000 (Reference)
65-69	-0.243	0.260	-0.932	0.351	0.784 (0.471-1.307)
70-74	-0.208	0.257	-0.809	0.418	0.812 (0.491-1.344)
≥75	0.292	0.236	1.234	0.217	1.339 (0.842-2.128)
Job					
Others					1.000 (Reference)
Farmers	-0.440	0.206	-2.141	0.032	0.644 (0.430-0.963)
Marital status					
Separated/divorced/widowed/unmarried					1.000 (Reference)
Married/cohabiting	-0.258	0.334	-0.773	0.440	0.773 (0.402-1.486)
Educational level					
Primary school and below					1.000 (Reference)
Junior middle school	0.097	0.193	0.503	0.615	1.102 (0.755-1.609)
Technical secondary/high school	0.263	0.263	1.002	0.316	1.301 (0.777-2.178)
College and above	0.488	0.311	1.569	0.117	1.629 (0.886-2.995)
Residential status					
Living alone					1.000 (Reference)
Living only with spouse	-0.412	0.347	-1.187	0.235	0.662 (0.335-1.308)
Living only with children	-0.255	0.315	-0.809	0.419	0.775 (0.418-1.437)
Living with spouse and children	-0.351	0.366	-0.961	0.337	0.704 (0.343-1.442)
Annual personal income, yuan					
＜4,999					1.000 (Reference)
5,000-9,999	0.437	0.217	2.013	0.044	1.548 (1.012-2.370)
10,000-19,999	0.510	0.266	1.919	0.055	1.666 (0.989-2.804)
≥20 000	0.987	0.225	4.388	<0.001	2.684 (1.727-4.171)

Abbreviations: Q1. Quartile 1; Q2. Quartile 2; Q3. Quartile 3; Q4. Quartile 4; HDL-C. high density lipoprotein cholesterol; HbA1c. hemoglobin A1c; HR: Hazards Ratio, CI: Confidence Interval; S.E. standard error;

Model 2: adjusted for sex, age at screening, marital status, educational level, job, residential status, and annual personal income.

Supplementary Table 9 Association between HbA1c/HDL-C ratio and cardiovascular disease risk by cox regression analyses of Model 3.

Variables	*β*	*S.E*	*Z*	*P*	HR (95%*CI*)
HbA1c/HDL-C ratio level					
Q1					1.000 (Reference)
Q2	-0.233	0.245	-0.952	0.341	0.792 (0.490-1.280)
Q3	0.442	0.216	2.044	0.041	1.556 (1.018-2.376)
Q4	0.740	0.206	3.582	<0.001	2.095 (1.398-3.140)
Gender					
Males					1.000 (Reference)
Females	0.157	0.149	1.053	0.292	1.169 (0.874-1.565)
Age at screening, years					
60-64					1.000 (Reference)
65-69	-0.267	0.263	-1.016	0.310	0.766 (0.458-1.281)
70-74	-0.223	0.258	-0.864	0.387	0.800 (0.483-1.326)
≥75	0.293	0.237	1.236	0.216	1.340 (0.842-2.133)
Job					
Others					1.000 (Reference)
Farmers	-0.421	0.208	-2.021	0.043	0.657 (0.437-0.987)
Marital status					
Separated/divorced/widowed/unmarried					1.000 (Reference)
Married/cohabiting	-0.260	0.335	-0.776	0.438	0.771 (0.400-1.487)
Educational level					
Primary school and below					1.000 (Reference)
Junior middle school	0.087	0.195	0.446	0.655	1.091 (0.745-1.597)
Technical secondary/high school	0.220	0.270	0.815	0.415	1.246 (0.734-2.117)
College and above	0.442	0.316	1.399	0.162	1.555 (0.838-2.889)
Residential status					
Living alone					1.000 (Reference)
Living only with spouse	-0.414	0.349	-1.187	0.235	0.661 (0.334-1.310)
Living only with children	-0.241	0.316	-0.763	0.446	0.786 (0.424-1.459)
Living with spouse and children	-0.345	0.368	-0.937	0.349	0.709 (0.345-1.457)
Annual personal income, yuan					
＜4,999					1.000 (Reference)
5,000-9,999	0.431	0.219	1.967	0.049	1.539 (1.002-2.363)
10,000-19,999	0.484	0.269	1.800	0.072	1.622 (0.958-2.747)
≥20 000	0.974	0.229	4.247	<0.001	2.649 (1.690-4.152)
Family history of coronary heart disease					
No					1.000 (Reference)
Yes	0.213	0.243	0.874	0.382	1.237 (0.768-1.994)
Family history of hypertension					
No					1.000 (Reference)
Yes	0.009	0.190	0.049	0.961	1.009 (0.695-1.466)
Family history of diabetes					

No					1.000 (Reference)
Yes	-0.001	0.256	-0.004	0.997	0.999 (0.605-1.650)

Abbreviations: Q1. Quartile 1; Q2. Quartile 2; Q3. Quartile 3; Q4. Quartile 4; HDL-C. high density lipoprotein cholesterol; HbA1c. hemoglobin A1c; HR: Hazards Ratio, CI: Confidence Interval; S.E. standard error;

Model 3, further adjusted for clinical variables (family history of coronary heart disease, family history of hypertension, family history of diabetes)

Supplementary Table 10 Association between HbA1c/HDL-C ratio and cardiovascular disease risk by cox regression analyses of Model 4.

Variables	*β*	*S.E*	*Z*	*P*	HR (95%*CI*)
HbA1c/HDL-C ratio level					
Q1					1.000 (Reference)
Q2	-0.188	0.240	-0.823	0.410	0.829 (0.502-1.414)
Q3	0.505	0.210	2.247	0.025	1.657 (1.098-2.501)
Q4	0.986	0.198	4.815	<0.001	2.680 (1.818-3.951)
Gender					
Males					1.000 (Reference)
Females	-0.063	0.140	-0.449	0.654	0.939 (0.715-1.235)
Age at screening, years					
60-64					1.000 (Reference)
65-69	-0.232	0.255	-0.909	0.363	0.793 (0.482-1.307)
70-74	-0.462	0.251	-1.836	0.066	0.630 (0.385-1.032)
≥75	-0.030	0.222	-0.134	0.894	0.971 (0.628-1.500)
Job					
Others					1.000 (Reference)
Farmers	-1.152	0.162	-7.111	<0.001	0.316 (0.230-0.434)
Marital status					
Separated/divorced/widowed/unmarried					1.000 (Reference)
Married/cohabiting	-0.282	0.173	-1.631	0.103	0.754 (0.537-1.059)
Educational level					
Primary school and below					1.000 (Reference)
Junior middle school	0.317	0.172	1.848	0.065	1.373 (0.981-1.922)
Technical secondary/high school	1.002	0.217	4.616	<0.001	2.724 (1.780-4.169)
College and above	1.352	0.258	5.236	<0.001	3.866 (2.330-6.412)
Residential status					
Living alone					1.000 (Reference)
Living only with spouse	-0.348	0.198	-1.754	0.079	0.706 (0.479-1.042)
Living only with children	-0.058	0.299	-0.195	0.845	0.943 (0.525-1.694)
Living with spouse and children	-0.266	0.231	-1.153	0.249	0.766 (0.488-1.205)
Annual personal income, yuan					
＜4,999					1.000 (Reference)
5,000-9,999	0.370	0.215	1.724	0.085	1.448 (0.951-2.204)
10,000-19,999	0.424	0.260	1.630	0.103	1.528 (0.918-2.544)
≥20 000	1.246	0.171	7.308	<0.001	3.477 (2.489-4.858)
Family history of coronary heart disease					
No					1.000 (Reference)
Yes	0.721	0.207	3.482	<0.001	2.057 (1.371-3.087)
Family history of hypertension					
No					1.000 (Reference)
Yes	0.510	0.154	3.317	<0.001	1.666 (1.232-2.252)
Family history of diabetes					

No					1.000 (Reference)
Yes	0.655	0.237	2.766	0.006	1.924 (1.210-3.060)
Smoking status					
No					1.000 (Reference)
Yes	0.113	0.190	0.594	0.553	1.120 (0.771-1.626)
Drinking status					
Non-drinker					1.000 (Reference)
Light drinking	0.345	0.223	1.546	0.122	1.412 (0.912-2.185)
Heavy drinking regularly	0.718	0.326	2.199	0.028	2.050 (1.081-3.887)
Exercise habits					
Regular exercise					1.000 (Reference)
Insufficient exercise	-0.038	0.175	-0.217	0.829	0.963 (0.684-1.356)
Dietary taste					
Moderate					1.000 (Reference)
Light taste	-0.537	0.212	-2.536	0.011	0.585 (0.386-0.885)
Salty taste	-0.609	0.268	-2.277	0.023	0.544 (0.322-0.919)
Dietary habits					
Balanced diet					1.000 (Reference)
Vegetarian-biased diet	-0.855	0.221	-3.874	<0.001	0.425 (0.276-0.655)
Meat-biased diet	0.281	0.456	0.616	0.538	1.325 (0.542-3.240)
Vegetable consumption frequency					
≤2 days per week					1.000 (Reference)
3-4 days per week	-0.401	0.215	-1.863	0.062	0.670 (0.440-1.021)
≥5 days per week	-1.121	0.364	-3.082	0.002	0.326 (0.160-0.665)
Fruit consumption frequency					
≤2 days per week					1.000 (Reference)
3-4 days per week	-1.180	0.213	-5.551	<0.001	0.307 (0.202-0.466)
≥5 days per week	-1.424	0.154	-9.270	<0.001	0.241 (0.178-0.325)

Abbreviations: Q1. Quartile 1; Q2. Quartile 2; Q3. Quartile 3; Q4. Quartile 4; HDL-C. high density lipoprotein cholesterol; HbA1c. hemoglobin A1c; HR: Hazards Ratio, CI: Confidence Interval; S.E. standard error;

Model 4: additionally adjusted for lifestyle factors (smoking status,drinking status, exercise habits, dietary taste, dietary patterns, vegetable consumption frequency, and fruit consumption frequency)

Supplementary Table 11 Association between HbA1c/HDL-C ratio and cardiovascular disease risk by cox regression analyses of Model 5.

Variables	*β*	*S.E*	*Z*	*P*	HR (95%*CI*)
HbA1c/HDL-C ratio level					
Q1					1.000 (Reference)
Q2	-0.294	0.253	-1.159	0.247	0.746 (0.454-1.225)
Q3	0.343	0.228	1.504	0.133	1.409 (0.901-2.204)
Q4	0.708	0.224	3.157	0.002	2.029 (1.308-3.149)
Gender					
Males					1.000 (Reference)
Females	0.148	0.173	0.855	0.393	1.160 (0.826-1.629)
Age at screening, years					
60-64					1.000 (Reference)
65-69	-0.323	0.269	-1.201	0.230	0.724 (0.428-1.226)
70-74	-0.317	0.264	-1.203	0.229	0.728 (0.434-1.221)
≥75	0.220	0.247	0.894	0.372	1.247 (0.769-2.022)
Job					
Others					1.000 (Reference)
Farmers	-0.229	0.212	-1.079	0.281	0.795 (0.525-1.205)
Marital status					
Separated/divorced/widowed/unmarried					1.000 (Reference)
Married/cohabiting	-0.254	0.338	-0.752	0.452	0.775 (0.400-1.504)
Educational level					
Primary school and below					1.000 (Reference)
Junior middle school	0.015	0.196	0.076	0.939	1.015 (0.691-1.490)
Technical secondary/high school	0.164	0.271	0.605	0.545	1.178 (0.692-2.005)
College and above	0.542	0.325	1.665	0.096	1.719 (0.908-3.252)
Residential status					
Living alone					1.000 (Reference)
Living only with spouse	-0.422	0.349	-1.210	0.226	0.656 (0.331-1.299)
Living only with children	-0.366	0.319	-1.146	0.252	0.694 (0.371-1.297)
Living with spouse and children	-0.446	0.369	-1.207	0.227	0.640 (0.310-1.321)
Annual personal income, yuan					
＜4,999					1.000 (Reference)
5,000-9,999	0.316	0.225	1.407	0.159	1.372 (0.883-2.133)
10,000-19,999	-0.024	0.290	-0.082	0.934	0.976 (0.553-1.723)
≥20 000	0.023	0.277	0.084	0.933	1.024 (0.595-1.761)
Family history of coronary heart disease					
No					1.000 (Reference)
Yes	0.126	0.246	0.512	0.609	1.134 (0.701-1.835)
Family history of hypertension					
No					1.000 (Reference)
Yes	-0.067	0.191	-0.349	0.727	0.935 (0.643-1.361)
Family history of diabetes					

No					1.000 (Reference)
Yes	-0.208	0.256	-0.812	0.417	0.812 (0.492-1.342)
Smoking status					
No					1.000 (Reference)
Yes	-0.405	0.248	-1.631	0.103	0.667 (0.410-1.085)
Drinking status					
Non-drinker					1.000 (Reference)
Light drinking	0.347	0.273	1.271	0.204	1.415 (0.829-2.416)
Heavy drinking regularly	0.821	0.377	2.180	0.029	2.273 (1.087-4.754)
Exercise habits					
Regular exercise					1.000 (Reference)
Insufficient exercise	0.448	0.198	2.265	0.024	1.565 (1.062-2.307)
Dietary taste					
Moderate					1.000 (Reference)
Light taste	0.223	0.303	0.736	0.462	1.250 (0.690-2.263)
Salty taste	-0.086	0.415	-0.207	0.836	0.917 (0.407-2.069)
Dietary habits					
Balanced diet					1.000 (Reference)
Vegetarian-biased diet	-0.937	0.371	-2.524	0.012	0.392 (0.189-0.811)
Meat-biased diet	1.099	0.591	1.859	0.063	3.002 (0.942-9.565)
Vegetable consumption frequency					
≤2 days per week					1.000 (Reference)
3-4 days per week	-0.064	0.256	-0.251	0.802	0.938 (0.567-1.550)
≥5 days per week	-1.028	0.394	-2.610	0.009	0.358 (0.165-0.774)
Fruit consumption frequency					
≤2 days per week					1.000 (Reference)
3-4 days per week	-0.637	0.301	-2.116	0.034	0.529 (0.293-0.954)
≥5 days per week	-1.310	0.247	-5.299	<0.001	0.270 (0.166-0.438)
Hypertension					
No					1.000 (Reference)
Yes	-0.192	0.160	-1.196	0.232	0.825 (0.603-1.130)
Overweight/obesity					
No					1.000 (Reference)
Yes	0.144	0.154	0.938	0.348	1.155 (0.855-1.561)

Abbreviations: Q1. Quartile 1; Q2. Quartile 2; Q3. Quartile 3; Q4. Quartile 4; HDL-C. high density lipoprotein cholesterol; HbA1c. hemoglobin A1c; HR: Hazards Ratio, CI: Confidence Interval; S.E. standard error;

Model 5: additionally adjusted for hypertension and overweight/obesity.

Supplementary Table 12 Association between HbA1c/HDL-C ratio and cardiovascular disease risk by cox regression analyses of Model 6.

Variables	*β*	*S.E*	*Z*	*P*	HR (95%*CI*)
HbA1c/HDL-C ratio level					
Q1					1.000 (Reference)
Q2	-0.304	0.254	-1.196	0.232	0.738 (0.449-1.214)
Q3	0.330	0.230	1.436	0.151	1.391 (0.887-2.181)
Q4	0.699	0.232	3.011	0.003	2.111 (1.276-3.170)
Gender					
Males					1.000 (Reference)
Females	0.151	0.176	0.857	0.392	1.162 (0.824-1.640)
Age at screening, years					
60-64					1.000 (Reference)
65-69	-0.325	0.269	-1.208	0.227	0.722 (0.426-1.224)
70-74	-0.316	0.264	-1.195	0.232	0.729 (0.435-1.224)
≥75	0.215	0.248	0.868	0.385	1.240 (0.763-2.017)
Job					
Others					1.000 (Reference)
Farmers	-0.229	0.212	-1.081	0.280	0.795 (0.524-1.205)
Marital status					
Separated/divorced/widowed/unmarried					1.000 (Reference)
Married/cohabiting	-0.263	0.339	-0.775	0.438	0.769 (0.395-1.495)
Educational level					
Primary school and below					1.000 (Reference)
Junior middle school	0.025	0.197	0.126	0.899	1.025 (0.697-1.507)
Technical secondary/high school	0.170	0.271	0.628	0.530	1.186 (0.697-2.018)
College and above	0.549	0.326	1.685	0.092	1.731 (0.914-3.277)
Residential status					
Living alone					1.000 (Reference)
Living only with spouse	-0.412	0.350	-1.177	0.239	0.662 (0.334-1.315)
Living only with children	-0.352	0.320	-1.101	0.271	0.703 (0.376-1.316)
Living with spouse and children	-0.444	0.370	-1.200	0.230	0.642 (0.311-1.325)
Annual personal income, yuan					
＜4,999					1.000 (Reference)
5,000-9,999	0.326	0.226	1.441	0.150	1.385 (0.889-2.157)
10,000-19,999	-0.013	0.293	-0.043	0.966	0.988 (0.556-1.753)
≥20 000	0.036	0.279	0.130	0.897	1.037 (0.601-1.790)
Family history of coronary heart disease					
No					1.000 (Reference)
Yes	-0.410	0.248	-1.652	0.098	0.664 (0.409-1.079)
Family history of hypertension					
No					1.000 (Reference)
Yes	0.355	0.272	1.303	0.193	1.426 (0.836-2.433)
Family history of diabetes	0.818	0.377	2.171	0.030	2.266 (1.083-4.742)

No					
Yes					1.000 (Reference)
Smoking status	0.447	0.198	2.257	0.024	1.564 (1.061-2.306)
No					
Yes					1.000 (Reference)
Drinking status	0.137	0.246	0.556	0.578	1.147 (0.708-1.857)
Non-drinker					
Light drinking					1.000 (Reference)
Heavy drinking regularly	-0.055	0.193	-0.284	0.777	0.947 (0.649-1.381)
Exercise habits					
Regular exercise					1.000 (Reference)
Insufficient exercise	-0.215	0.261	-0.823	0.410	0.806 (0.483-1.346)
Dietary taste					
Moderate					1.000 (Reference)
Light taste	0.222	0.303	0.733	0.463	1.248 (0.690-2.259)
Salty taste	-0.090	0.417	-0.216	0.829	0.914 (0.403-2.071)
Dietary habits					
Balanced diet					1.000 (Reference)
Vegetarian-biased diet	-0.915	0.373	-2.455	0.014	0.400 (0.193-0.831)
Meat-biased diet	1.090	0.595	1.833	0.067	2.975 (0.927-9.547)
Vegetable consumption frequency					
≤2 days per week					1.000 (Reference)
3-4 days per week	-0.061	0.257	-0.237	0.813	0.941 (0.569-1.557)
≥5 days per week	-1.012	0.394	-2.568	0.010	0.363 (0.168-0.787)
Fruit consumption frequency					
≤2 days per week					1.000 (Reference)
3-4 days per week	-0.650	0.302	-2.155	0.031	0.522 (0.289-0.943)
≥5 days per week	-1.309	0.248	-5.271	<0.001	0.270 (0.166-0.439)
Hypertension					
No					1.000 (Reference)
Yes	-0.192	0.162	-1.188	0.235	0.825 (0.601-1.133)
Diabetes					
No					1.000 (Reference)
Yes	0.064	0.191	0.332	0.740	1.066 (0.732-1.551)
Dyslipidemia					
No					1.000 (Reference)
Yes	-0.090	0.164	-0.551	0.582	0.914 (0.663-1.260)
Overweight/obesity					
No					1.000 (Reference)
Yes	0.157	0.155	1.015	0.310	1.170 (0.864-1.586)

Abbreviations: Q1. Quartile 1; Q2. Quartile 2; Q3. Quartile 3; Q4. Quartile 4; HDL-C. high density lipoprotein cholesterol; HbA1c. hemoglobin A1c; HR: Hazards Ratio, CI: Confidence Interval; S.E. standard error;

Model 6, further adjusted for diabetes, dyslipidemia.

Supplementary Table 13 Association between HbA1c/HDL-C ratio and cardiovascular disease risk by cox regression analyses of Model 6[a].

Variables	*β*	*S.E*	*Z*	*P*	HR (95%*CI*)
HbA1c/HDL-C ratio level					
Q1					1.000 (Reference)
Q2	-0.346	0.251	-1.380	0.168	0.708 (0.433- 1.157)
Q3	0.256	0.224	1.142	0.254	1.292 (0.832- 2.005)
Q4	0.618	0.225	2.751	0.006	1.855 (1.194-2.880)
Age at screening, years					
60-64					1.000 (Reference)
65-69	-0.387	0.265	-1.461	0.144	0.679 (0.404-1.141)
70-74	-0.350	0.260	-1.345	0.179	0.705 (0.424- 1.173)
≥75	0.128	0.240	0.536	0.592	1.137 (0.711-1.819)
Job					
Others					1.000 (Reference)
Farmers	-0.225	0.209	-1.080	0.280	0.798 (0.530-1.202)
Marital status					
Separated/divorced/widowed/unmarried					1.000 (Reference)
Married/cohabiting	-0.582	0.187	-3.121	0.002	0.559 (0.387-0.805)
Educational level					
Primary school and below					1.000 (Reference)
Junior middle school	-0.013	0.193	-0.067	0.947	0.987 (0.676-1.441)
Technical secondary/high school	0.093	0.266	0.351	0.726	1.098 (0.652-1.849)
College and above	0.479	0.318	1.507	0.132	1.614 (0.866-3.009)
Annual personal income, yuan					
＜4,999					1.000 (Reference)
5,000-9,999	0.324	0.224	1.446	0.148	1.382 (0.891- 2.143)
10,000-19,999	-0.007	0.289	-0.025	0.980	0.993 (0.563-1.750)
≥20 000	0.050	0.274	0.183	0.855	1.052 (0.614-1.800)
Family history of coronary heart disease					
No					1.000 (Reference)
Yes	0.163	0.244	0.666	0.506	1.177 (0.729-1.899)
Family history of hypertension					
No					1.000 (Reference)
Yes	-0.051	0.192	-0.267	0.789	0.950 (0.652-1.384)
Family history of diabetes					
No					1.000 (Reference)
Yes	-0.148	0.259	-0.572	0.567	0.862 (0.519-1.433)
Hypertension					
No					1.000 (Reference)
Yes	-0.161	0.159	-1.016	0.310	0.851 (0.623-1.162)
Diabetes					
No					1.000 (Reference)
Yes	0.095	0.190	0.499	0.617	1.099 (0.758-1.594)

Dyslipidemia					
No					1.000 (Reference)
Yes	-0.079	0.161	-0.491	0.623	0.924 (0.673-1.268)
Overweight/obesity					
No					1.000 (Reference)
Yes	0.196	0.154	1.274	0.203	1.217 (0.900-1.645)
Dietary habits					
Balanced diet					1.000 (Reference)
Vegetarian-biased diet	-0.766	0.286	-2.681	0.007	0.465 (0.266-0.814)
Meat-biased diet	1.011	0.487	2.077	0.038	2.747 (1.059-7.129)
Vegetable consumption frequency					
≤2 days per week					1.000 (Reference)
3-4 days per week	0.016	0.236	0.069	0.945	1.016 (0.640-1.615)
≥5 days per week	-0.917	0.388	-2.365	0.018	0.400 (0.187-0.855)
Fruit consumption frequency					
≤2 days per week					1.000 (Reference)
3-4 days per week	-0.640	0.295	-2.169	0.030	0.527 (0.295-0.940)
≥5 days per week	-1.192	0.238	-5.020	<.001	0.304 (0.191-0.483)

Abbreviations: Q1. Quartile 1; Q2. Quartile 2; Q3. Quartile 3; Q4. Quartile 4; HDL-C. high density lipoprotein cholesterol; HbA1c. hemoglobin A1c; HR: Hazards Ratio, CI: Confidence Interval; S.E. standard error;

Model 6[a]: Based on Model 6, only variables with $P < 0.05$ in univariate analysis were adjusted for, including age at screening, years, marital status, educational level, job, annual personal income, family history of coronary heart disease, family history of hypertension, family history of diabetes, dietary habits, vegetable consumption frequency, fruit consumption frequency, hypertension, diabetes, dyslipidemia, overweight/obesity.

Supplementary Table 14 Association between HbA1c/HDL-C ratio and cardiovascular disease risk by cox regression analyses stratified by event time.

HbA1c/HDL-C	＜3 years		3-5.99 years		≥6 years	
	HR(95%*CI*)	*P*	HR(95%*CI*)	*P*	HR(95%*CI*)	*P*
Q1	1.000 (Reference)		1.000 (Reference)		1.000 (Reference)	
Q2	0.622 (0.177-2.190)	0.459	0.853 (0.368-1.974)	0.710	1.220 (0.461-3.232)	0.689
Q3	1.429 (0.472-4.329)	0.528	1.633 (0.784-3.399)	0.19	1.150 (0.376-3.516)	0.807
Q4	4.039 (1.379 -11.825)	0.011	3.057 (1.574-5.939)	<0.001	1.454 (0.441-4.789)	0.538

Abbreviations: Q1. Quartile 1; Q2. Quartile 2; Q3. Quartile 3; Q4. Quartile 4. CI. confidence interval; HR. hazard ratio; CVD.cardiovascular disease; HDL-C.high-density lipoprotein cholesterol; HbA1c, hemoglobin A1c

Supplementary Table 15 The association between HbA1c, HDL-C or HbA1c/HDL-C level and the risk of cardiovascular disease.

Variables	HR (95%*CI*)	*P*	HR (95%*CI*)	*P*	HR (95%*CI*)	*P*
Gender						
Males	1.000 (Reference)		1.000 (Reference)		1.000 (Reference)	
Females	1.018 (0.726-1.426)	0.920	1.095 (0.777-1.541)	0.605	0.939 (0.715-1.235)	0.654
Age at screening, years						
60-64	1.000 (Reference)		1.000 (Reference)		1.000 (Reference)	
65-69	0.695 (0.410-1.177)	0.176	0.688 (0.407-1.163)	0.163	0.793 (0.482-1.307)	0.363
70-74	0.741 (0.442-1.241)	0.254	0.757 (0.452-1.268)	0.290	0.630 (0.385-1.032)	0.066
≥75	1.264 (0.780-2.046)	0.342	1.275 (0.787-2.064)	0.324	0.971 (0.628-1.500)	0.894
Job						
Others	1.000 (Reference)		1.000 (Reference)		1.000 (Reference)	
Farmers	0.751 (0.493-1.142)	0.180	0.784 (0.518-1.187)	0.251	0.316 (0.230-0.434)	<.001
Marital status						
Separated/divorced/widowed/unmarried	1.000 (Reference)		1.000 (Reference)		1.000 (Reference)	
Married/cohabiting	0.828 (0.427-1.609)	0.578	0.858 (0.442-1.665)	0.651	0.754 (0.537-1.059)	0.103
Educational level						
Primary school and below	1.000 (Reference)		1.000 (Reference)		1.000 (Reference)	
Junior middle school	0.999 (0.680-1.467)	0.995	1.003 (0.684-1.469)	0.989	1.373 (0.981-1.922)	0.065
Technical secondary/high school	1.212 (0.711-2.067)	0.479	1.232 (0.724-2.097)	0.442	2.724 (1.780-4.169)	<.001
College and above	1.418 (0.744-2.703)	0.288	1.545 (0.816-2.927)	0.182	3.866 (2.330-6.412)	<.001
Residential status						
Living alone	1.000 (Reference)		1.000 (Reference)		1.000 (Reference)	
Living only with spouse	0.621 (0.316-1.220)	0.167	0.594 (0.300-1.177)	0.135	0.706 (0.479-1.042)	0.079
Living only with children	0.705 (0.380-1.308)	0.268	0.701 (0.376-1.308)	0.264	0.943 (0.525-1.694)	0.845
Living with spouse and children	0.587 (0.287-1.198)	0.143	0.566 (0.274-1.166)	0.123	0.766 (0.488-1.205)	0.249

Annual personal income, yuan						
＜4,999	1.000 (Reference)		1.000 (Reference)		1.000 (Reference)	
5,000-9,999	1.390 (0.895-2.160)	0.143	1.396 (0.901-2.162)	0.135	1.448 (0.951-2.204)	0.085
10,000-19,999	0.896 (0.505-1.588)	0.706	0.913 (0.515-1.619)	0.755	1.528 (0.918-2.544)	0.103
≥20 000	1.089 (0.633-1.875)	0.758	1.080 (0.627-1.858)	0.782	3.477 (2.489-4.858)	<.001
Family history of coronary heart disease						
No	1.000 (Reference)		1.000 (Reference)		1.000 (Reference)	
Yes	1.169 (0.718-1.904)	0.531	1.159 (0.716-1.875)	0.549	2.057 (1.371-3.087)	<.001
Family history of hypertension						
No	1.000 (Reference)		1.000 (Reference)		1.000 (Reference)	
Yes	0.888 (0.610-1.292)	0.534	0.904 (0.620-1.318)	0.598	1.666 (1.232-2.252)	<.001
Family history of diabetes						
No	1.000 (Reference)		1.000 (Reference)		1.000 (Reference)	
Yes	0.814 (0.489-1.355)	0.429	0.791 (0.475-1.318)	0.369	1.924 (1.210-3.060)	0.006
Hypertension						
No	1.000 (Reference)		1.000 (Reference)		1.000 (Reference)	
Yes	0.872 (0.637-1.193)	0.391	0.819 (0.598-1.122)	0.213	1.155 (0.867-1.538)	0.324
Diabetes						
No	——		1.000 (Reference)		——	
Yes	——		1.250 (0.873-1.791)	0.223	——	
Dyslipidemia						
No	1.000 (Reference)		——		——	
Yes	1.013 (0.739-1.391)	0.934	——		——	
Overweight/obesity						
No	1.000 (Reference)		1.000 (Reference)		1.000 (Reference)	
Yes	1.324 (0.985-1.778)	0.063	1.155 (0.855-1.560)	0.349	1.409 (1.072-1.851)	0.014

Smoking status						
No	1.000 (Reference)		1.000 (Reference)		1.000 (Reference)	
Yes	0.720 (0.446-1.164)	0.181	0.688 (0.425-1.112)	0.127	1.120 (0.771-1.626)	0.553
Drinking status						
Non-drinker	1.000 (Reference)		1.000 (Reference)		1.000 (Reference)	
Light drinking	1.400 (0.825-2.375)	0.213	1.464 (0.864-2.481)	0.157	1.412 (0.912-2.185)	0.122
Heavy drinking regularly	1.977 (0.947-4.127)	0.070	2.220 (1.059-4.653)	0.035	2.050 (1.081-3.887)	0.028
Exercise habits						
Regular exercise	1.000 (Reference)		1.000 (Reference)		1.000 (Reference)	
Insufficient exercise	1.586 (1.077-2.334)	0.019	1.592 (1.082-2.344)	0.018	0.963 (0.684-1.356)	0.829
Dietary taste						
Moderate	1.000 (Reference)		1.000 (Reference)		1.000 (Reference)	
Light taste	1.293 (0.711-2.352)	0.399	1.213 (0.673-2.187)	0.521	0.585 (0.386-0.885)	0.011
Salty taste	0.874 (0.386-1.979)	0.747	0.859 (0.383-1.927)	0.712	0.544 (0.322-0.919)	0.023
Dietary habits						
Balanced diet	1.000 (Reference)		1.000 (Reference)		1.000 (Reference)	
Vegetarian-biased diet	0.416 (0.201-0.857)	0.017	0.450 (0.219-0.925)	0.030	0.425 (0.276-0.655)	<.001
Meat-biased diet	3.263 (1.017-10.467)	0.047	2.971 (0.930-9.491)	0.066	1.325 (0.542-3.240)	0.538
Vegetable consumption frequency						
≤2 days per week	1.000 (Reference)		1.000 (Reference)		1.000 (Reference)	
3-4 days per week	0.868 (0.522-1.441)	0.583	0.919 (0.557-1.515)	0.740	0.670 (0.440-1.021)	0.062
≥5 days per week	0.391 (0.181-0.843)	0.017	0.386 (0.180-0.828)	0.014	0.326 (0.160-0.665)	0.002
Fruit consumption frequency						
≤2 days per week	1.000 (Reference)		1.000 (Reference)		1.000 (Reference)	
3-4 days per week	0.496 (0.273-0.900)	0.021	0.469 (0.260-0.847)	0.012	0.307 (0.202-0.466)	<.001
≥5 days per week	0.249 (0.153-0.404)	<0.001	0.266 (0.164-0.432)	<0.001	0.241 (0.178-0.325)	<0.001

HbA1c	1.094 (0.969-1.236)	0.145				
HDL-C			0.559 (0.380-0.822)	0.003		
HbA1c/HDL-C ratio					1.291 (1.183-1.409)	<0.001

Abbreviations:HbA1c. Glycated hemoglobin A1c; HDL-C. High-density lipoprotein cholesterol; HR. Hazard ratio; CI. Confidence interval

Supplementary Table 16 Subgroup analyses for the association between the HbA1c/HDL-C ratio and incident CVD stratified by the inflection point identified via restricted cubic spline analysis.

HbA1c/HDL-C	Total	CVD(%)	HR (95%*CI*)	*P*
＜4.83	1587	157(9.89)	1.441 (1.138-1.825)	0.002
≥4.83	288	50(17.36)	1.167 (0.864-1.576)	0.315

Abbreviations: HbA1c. Glycated hemoglobin A1c; HDL-C. High-density lipoprotein cholesterol; HR. Hazard ratio; CI. Confidence interval

Supplementary Table 17 Baseline characteristic comparison between the training set and test set grouped for the Cox regression prediction model.

Variables	Total (n=1,875)	test (n=563)	train (n=1312)	Statistic	*P*
Gender				3.345	0.067
Males	913 (48.69)	256 (45.47)	657 (50.08)		
Females	962 (51.31)	307 (54.53)	655 (49.92)		
Age at screening, years				2.280	0.516
60-64	282 (15.04)	88 (15.63)	194 (14.79)		
65-69	419 (22.35)	129 (22.91)	290 (22.10)		
70-74	531 (28.32)	167 (29.66)	364 (27.74)		
≥75	643 (34.29)	179 (31.79)	464 (35.37)		
Marital status				1.342	0.247
Separated/divorced/widowed/unmarried	270 (14.40)	73 (12.97)	197 (15.02)		
Married/cohabiting	1605 (85.60)	490 (87.03)	1115 (84.98)		
Educational level				3.130	0.372
Primary school and below	1163 (62.03)	343 (60.92)	820 (62.50)		
Junior middle school	499 (26.61)	152 (27.00)	347 (26.45)		
Technical secondary/high school	150 (8.00)	43 (7.64)	107 (8.16)		
College and above	63 (3.36)	25 (4.44)	38 (2.90)		
Job				1.034	0.309
Others	225 (12.00)	61 (10.83)	164 (12.50)		
Farmers	1650 (88.00)	502(89.17)	1148 (87.60)		
Residential status				3.081	0.379
Living alone	215 (11.47)	55 (9.77)	160 (12.20)		
Living only with spouse	1074 (57.28)	336 (59.68)	738 (56.25)		
Living only with children	114 (6.08)	35 (6.22)	79 (6.02)		
Living with spouse and children	472 (25.17)	137 (24.33)	335 (25.53)		
Annual personal income, yuan				2.915	0.405
＜4,999	723 (38.56)	208 (36.94)	515 (39.25)		
5,000-9,999	421 (22.45)	133 (23.62)	288 (21.95)		
10,000-19,999	206 (10.99)	55 (9.77)	151 (11.51)		
≥20 000	525 (28.00)	167 (29.66)	358 (27.29)		
Family history of coronary heart disease				0.994	0.319
No	1764 (94.08)	525 (93.25)	1239 (94.44)		
Yes	111 (5.92)	38 (6.75)	73 (5.56)		
Family history of hypertension				0.341	0.559
No	1527 (81.44)	454 (80.64)	1073 (81.78)		
Yes	348 (18.56)	109 (19.36)	239 (18.22)		
Family history of diabetes				1.011	0.315
No	1761 (93.92)	524 (93.07)	1237 (94.28)		
Yes	114 (6.08)	39 (6.93)	75 (5.72)		
Hypertension				0.082	0.775
No	822 (43.84)	244 (43.34)	578 (44.05)		

Yes	1053 (56.16)	319 (56.66)	734 (55.95)		
Diabetes				0.012	0.914
No	1621 (86.45)	486 (86.32)	1135 (86.51)		
Yes	254 (13.55)	77 (13.68)	177 (13.49)		
Dyslipidemia				0.013	0.908
No	1319 (70.35)	395 (70.16)	924 (70.43)		
Yes	556 (29.65)	168 (29.84)	388 (29.57)		
Overweight/obesity				0.145	0.703
No	1140 (60.80)	346 (61.46)	794 (60.52)		
Yes	735 (39.20)	217 (38.54)	518 (39.48)		
Smoking status				0.894	0.344
No	1586 (84.59)	483 (85.79)	1103 (84.07)		
Yes	289 (15.41)	80 (14.21)	209 (15.93)		
Drinking status				0.104	0.949
Non-drinker	1641 (87.52)	494 (87.74)	1147 (87.42)		
Light drinking	184 (9.81)	55 (9.77)	129 (9.83)		
Heavy drinking regularly	50 (2.67)	14 (2.49)	36 (2.74)		
Exercise habits				0.188	0.664
Regular exercise	1514 (80.75)	458 (81.35)	1056 (80.49)		
Insufficient exercise	361 (19.25)	105 (18.65)	256 (19.51)		
Dietary taste				2.309	0.315
Moderate	1473 (78.56)	437 (77.62)	1036 (78.96)		
Light taste	274 (14.61)	80 (14.21)	194 (14.79)		
Salty taste	128 (6.83)	46 (8.17)	82 (6.25)		
Dietary habits				1.998	0.368
Balanced diet	1569 (83.68)	475 (84.37)	1094 (83.38)		
Vegetarian-biased diet	287 (15.31)	80 (14.21)	207 (15.78)		
Meat-biased diet	19 (1.01)	8 (1.42)	11 (0.84)		
Vegetable consumption frequency				0.789	0.674
≤2 days per week	1413 (75.36)	430 (76.38)	983 (74.92)		
3-4 days per week	315 (16.80)	88 (15.63)	227 (17.30)		
≥5 days per week	147 (7.84)	45 (7.99)	102 (7.77)		
Fruit consumption frequency				0.927	0.629
≤2 days per week	496 (26.45)	144 (25.58)	352 (26.83)		
3-4 days per week	269 (14.35)	87 (15.45)	182 (13.87)		
≥5 days per week	1110 (59.20)	332 (58.97)	778 (59.30)		
HbA1c/HDL-C ratio level				1.898	0.594
Q1	469 (25.01)	148 (26.29)	321 (24.47)		
Q2	473 (25.23)	147 (26.11)	326 (24.85)		
Q3	469 (25.01)	139 (24.69)	330 (25.15)		
Q4	464 (24.75)	129 (22.91)	335 (25.53)		

Abbreviations: Q1. Quartile 1; Q2. Quartile 2; Q3. Quartile 3; Q4. Quartile 4; HDL-C. high density lipoprotein; HbA1c. hemoglobin A1c.

Supplementary Table 18 Quantitative net benefit and net drift values of time-dependent DCA for Model A and Model B.

Event Time	Cohort	Model	Peak Net Benefit	Useful Risk Threshold Range	NB at 20% threshold	NB at 30% threshold
2-year	Train	A	0.000	No valid range	0.000	0.000
2-year	Test	A	0.000	No valid range	0.000	0.000
2-year	Train	B	0.000	No valid range	0.000	0.000
2-year	Test	B	0.000	No valid range	0.000	0.000
3-year	Train	A	0.041	0.22–0.44	0.030	0.041
3-year	Test	A	0.039	0.23–0.43	0.028	0.039
3-year	Train	B	0.040	0.22–0.43	0.031	0.042
3-year	Test	B	0.038	0.23–0.42	0.032	0.041
4-year	Train	A	0.132	0.15–0.61	0.118	0.132
4-year	Test	A	0.128	0.16–0.60	0.115	0.128
4-year	Train	B	0.130	0.15–0.63	0.120	0.136
4-year	Test	B	0.129	0.16–0.61	0.116	0.131
5-year	Train	A	0.186	0.12–0.69	0.171	0.186
5-year	Test	A	0.183	0.13–0.68	0.169	0.183
5-year	Train	B	0.187	0.12–0.70	0.172	0.189
5-year	Test	B	0.185	0.13–0.68	0.171	0.188
6-year	Train	A	0.179	0.11–0.66	0.165	0.179
6-year	Test	A	0.176	0.12–0.65	0.163	0.176
6-year	Train	B	0.183	0.11–0.68	0.168	0.189
6-year	Test	B	0.178	0.12–0.68	0.166	0.182
7-year	Train	A	0.064	0.10–0.35	0.064	0.039
7-year	Test	A	0.061	0.10–0.34	0.061	0.037
7-year	Train	B	0.068	0.10–0.35	0.068	0.041
7-year	Test	B	0.063	0.10–0.35	0.063	0.039

Abbreviations: NB. Net Benefit; DCA. decision curve analysis.

Supplementary Table 19 ROC analyses of different metabolic indexes for incident CVD events.

Index	AUC (95%*CI*)	Accuracy (95%*CI*)	Sensitivity (95%*CI*)	Specificity (95%*CI*)	PPV (95%*CI*)	NPV (95%*CI*)	Cut off
FBG	0.504 (0.460-0.547)	0.815 (0.797-0.833)	0.896 (0.882 - 0.911)	0.164 (0.114 - 0.215)	0.896 (0.882 - 0.911)	0.164 (0.114 - 0.215)	6.250
HbA1c	0.573 (0.531-0.614)	0.616 (0.594-0.638)	0.629 (0.606 - 0.653)	0.507 (0.439 - 0.575)	0.911 (0.895 - 0.928)	0.145 (0.120 - 0.171)	5.650
HDL-C	0.599 (0.558-0.640)	0.396 (0.374-0.418)	0.393 (0.369 - 0.416)	0.420 (0.353 - 0.488)	0.845 (0.820 - 0.871)	0.079 (0.063 - 0.095)	1.495
HbA1c/HDL-C	0.636 (0.564-0.647)	0.573 (0.550-0.595)	0.564 (0.540 - 0.588)	0.643 (0.577 - 0.708)	0.927 (0.911 - 0.943)	0.155 (0.130 - 0.179)	3.571
TyG	0.482 (0.442-0.522)	0.526 (0.504-0.549)	0.539 (0.515 - 0.563)	0.425 (0.358 - 0.492)	0.883 (0.863 - 0.903)	0.103 (0.082 - 0.123)	1.245
TG/HDL-C	0.560 (0.520-0.601)	0.498 (0.475-0.521)	0.482 (0.458 - 0.506)	0.628 (0.562 - 0.694)	0.913 (0.894 - 0.931)	0.131 (0.110 - 0.152)	0.775
non-HDL-C	0.529 (0.488-0.570)	0.443 (0.420-0.465)	0.435 (0.411 - 0.459)	0.502 (0.434 - 0.571)	0.876 (0.853 - 0.898)	0.099 (0.081 - 0.118)	2.905
AIP	0.560 (0.520-0.601)	0.516 (0.493-0.539)	0.505 (0.481 - 0.529)	0.604 (0.537 - 0.670)	0.911 (0.893 - 0.930)	0.132 (0.110 - 0.153)	-0.095

Abbreviations: AUC. area under the curve; CI. confidence interval; ROC. receiver operating characteristic; FBG. fasting blood glucose；HbA1c. glycated hemoglobin A1c；HDL-C. high-density lipoprotein cholesterol; HbA1c/HDL-C. glycated hemoglobin A1c to high-density lipoprotein cholesterol ratio；TyG. triglyceride-glucose index；TG/HDL-C. triglyceride to high-density lipoprotein cholesterol ratio；non-HDL-C. non-high-density lipoprotein cholesterol；AIP. atherogenic Index of plasma

Supplementary Table 20 Subgroup and interaction analyses of HbA1c/HDL-C ratio and cardiovascular disease risk.

Variables	Q1	Q2		Q3		Q4		P for interaction
		HR(95%*CI*)	*P*	*HR*(95%*CI*)	*P*	*HR*(95%*CI*)	*P*	
Gender								0.338
Males	1.000 (Reference)	2.026（0.873–4.699）	0.100	1.390（0.690–2.802）	0.357	1.779（0.838–3.774）	0.133	
Females	1.000 (Reference)	0.704（0.330–1.499）	0.362	1.909（0977–3.729）	0.058	2.032（0.992–4.161）	0.053	
Age at screening, years								0.568
55-64	1.000 (Reference)	——		——		——		
65-74	1.000 (Reference)	1.080（0.398–2.933）	0.880	2.026（0.969–4.236）	0.061	3.124（1.315–7.422）	0.010	
≥75	1.000 (Reference)	1.667（0.792–3.511）	0.178	1.576（0.782–3.177）	0.203	2.508（1.217–5.167）	0.013	
Marital status								0.824
Separated/divorced/widowed/unmarried	1.000 (Reference)	2.205（0.401–12.122）	0.363	0.661（0.181–2.412）	0.531	2.063（0.395–10.766）	0.390	
Married/cohabiting	1.000 (Reference)	1.131（0614–2.082）	0.693	1.718（1.017–2.903）	0.043	1.981（1.117–3.512）	0.019	
Educational level								0.904
Primary school and below	1.000 (Reference)	0.775（0.375–1.599）	0.490	1.302（0.689–2.461）	0.416	1.452（0.751–2.809）	0.268	
Junior middle school	1.000 (Reference)	3.169（0722–13.903）	0.126	4.429（1.428–13.742）	0.010	4.855（2.982–7.399）	0.001	
Technical secondary/high school	1.000 (Reference)	——		——		——		
College and above	1.000 (Reference)	——		——		——		
Job								0.999
Others		1.536(0.198-11.913)	0.681	0.992(0.193-5.093)	0.993	3.614(0.468-27.920)	0.218	
Farmers		1.065(0.603-1.880)	0.829	1.630(0.995-2.671)	0.052	1.780(1.054-3.004)	0.031	
Annual personal income, yuan								0.583
＜4,999	1.000 (Reference)	1.295(0.590-2.839)	0.303	1.747(0.653-4.676)	0.519	1.131(0.529-2.420)	0.287	
5,000-9,999	1.000 (Reference)	0.147(0.017-1.248)	0.079	0.301(0.064-1.412)	0.128	0.504(0.073-3.488)	0.487	
10,000-19,999	1.000 (Reference)	——		——		——		

≥20 000	1.000 (Reference)	2.673(0.942-6.858）	0.215	1.2440(.524-2.951)	0.621	3.098（1.229-7.810)	0.017	
Family history of coronary heart disease								0.948
No	1.000 (Reference)	1.055(0.599-1.858)	0.852	1.531(0.953-2.459)	0.078	1.962(1.189-3.238)	0.008	
Yes	1.000 (Reference)	——		——		——		
Family history of hypertension								0.607
No	1.000 (Reference)	1.087(0.588-2.010)	0.791	1.709(1.029-2.837)	0.038	1.981(1.157-3.391)	0.013	
Yes	1.000 (Reference)	1.343(0.374-4.824)	0.651	1.434(0.446-4.616)	0.545	3.109(0.815-11.859)	0.097	
Family history of diabetes								0.431
No	1.000 (Reference)	0.999(0.577-1.730)	0.997	1.696(1.073-2.682)	0.024	2.013(1.238-3.275)	0.003	
Yes	1.000 (Reference)	——		——		——		
Hypertension								0.541
No	1.000 (Reference)	1.090（0.107–2.918）	0.864	2.630（1.176–5.880）	0.018	2.802（1.180–6.657）	0.020	
Yes	1.000 (Reference)	1.306（0.690–2.466）	0.410	1.412（0.767–2.601）	0.268	2.080（0.067–4.504）	0.369	
Diabetes								0.434
No	1.000 (Reference)	1.071（0.0.617–1.862）	0.807	1.576（0.977–2.541）	0.062	1.754（1.023–3.008）	0.041	
Yes	1.000 (Reference)	0.858（0.075–9.867）	0.902	1.761（0.178–17.464）	0.629	3.206（0.432–23.768）	0.254	
Dyslipidemia								0.551
No	1.000 (Reference)	1.281（0.671–2443）	0.453	1.629（0.946–2.807）	0.079	1.896（0.995–6.612）	0.052	
Yes	1.000 (Reference)	1.168（0.440–3.101）	0.756	2.287（0.879–5.953）	0.090	2.342（0.932–5.886）	0.070	
Overweight/obesity								0.375
No	1.000 (Reference)	1.479（0.733–2.985）	0.275	2.606（1.354–5.013）	0.004	2.619（1.296–5.290）	0.007	
Yes	1.000 (Reference)	0.991（0.376–2.614）	0.985	1.344（0.620–2.911）	0.454	1.524（0.682–3.406）	0.305	
Smoking status								0.962
No	1.000 (Reference)	1.067（0.607–1.878）	0.821	1.644（0.009–2.678）	0.046	2.221（1.294–3.811）	0.004	
Yes	1.000 (Reference)	0.235（0.013–4.264）	0.328	1.115（0125–9.944）	0.922	0.988（0.119–8.170）	0.991	
Drinking status								0.064

Non-drinker	1.000 (Reference)	1.063（0.608–1.860）	0.829	1.766（1.069–2.918）	0.026	2.067（1.219–3.503）	0.007	
Light drinking	1.000 (Reference)	——		——		——		
Heavy drinking regularly	1.000 (Reference)	——		——		——		
Dietary habits								0.845
Balanced diet	1.000 (Reference)	1.094(0.613-1.951)	0.761	1.618(0.982-2.665)	0.059	1.964(0.342-3.379)	0.365	
Vegetarian-biased diet	1.000 (Reference)	0.009(0.001-7.099)	0.166	6.211(.011-34.314)	0.571	0.004(0.001-4.109）	0.120	
Meat-biased diet	1.000 (Reference)	——		——		——		
Vegetable consumption frequency								0.095
≤2 days per week	1.000 (Reference)	0.973(0.542-1.746)	0.926	1.860(1.128-3.067)	0.015	2.035(1.201-3.448)	0.008	
3-4 days per week	1.000 (Reference)	0.323(0.006-17.803)	0.580	0.668(0.057-7.883)	0.748	18.240(0.952-349.574)	0.054	
≥5 days per week	1.000 (Reference)	——		——		——		
Fruit consumption frequency								0.749
≤2 days per week	1.000 (Reference)	1.169(0.466-2.934)	0.740	1.407(0.575-3.440)	0.454	2.192(0.885-5.432)	0.090	
3-4 days per week	1.000 (Reference)	0.270(0.016-4.594)	0.366	0.105(0.004-2.456)	0.101	9.945(0.658-12.280)	0.365	
≥5 days per week	1.000 (Reference)	1.024(0.439-2.390)	0.956	1.479(0.727-3.006)	0.280	1.607(0.677-3.814)	0.282	

——. No results were output from the model;Abbreviations: Q1. Quartile 1; Q2. Quartile 2; Q3. Quartile 3; Q4. Quartile 4; HR: Hazards Ratio, CI: Confidence Interval; S,E. standard error. All models were for gender, age at screening, years, marital status, educational level, job, residential status, annual personal income, family history of coronary heart disease, family history of hypertension, family history of diabetes, hypertension, diabetes , dyslipidemia, overweight/obesity, smoking status, drinking status, exercise habits, dietary taste, dietary habits, vegetable consumption frequency, fruit consumption frequency.

Supplementary Table 21 The HbA1c/HDL-C and mortality in competing risk analyses.

HbA1c/HDL-C	Model 7		Model 8	
	sHR(95%*CI*)	*P*	*sHR*(95%*CI*)	*P*
Q1	1.000 (Reference)		1.000 (Reference)	
Q2	0.754(0.473-1.204)	0.238	0.645(0.389-1.068)	0.088
Q3	1.633(1.086-2.457)	0.019	1.388(0.892-2.160)	0.146
Q4	2.689(1.852-3.906)	＜0.001	2.007(1.279-3.152)	0.002

Model 7: unadjusted crude model;

Model 8:fully adjusted model, with covariates consistent with those adopted in Model 6

Abbreviations: Q1. Quartile 1; Q2. Quartile 2; Q3. Quartile 3; Q4. Quartile 4. CI. confidence interval; sHR. subdistribution hazard ratio.

Supplementary Table 22 Sensitivity analyses for the association between HbA1c/HDL-C level and cardiovascular disease risk.

HbA1c/HDL-C	Model 9				Model 10			
	Total (n=1180)	CVD (n=107)	*HR*(95%*CI*)	*P*	Total (n=410)	CVD (n=24)	*HR*(95%*CI*)	*P*
Q1	335 (28.39)	23 (6.87)	1.000 (Reference)		141 (34.39)	7 (4.96)	1.000 (Reference)	
Q2	343 (29.07)	19 (5.54)	0.782 (0.414-1.486)	0.442	126 (30.73)	2 (1.59)	1.499 (0.549-4.091)	0.430
Q3	314 (26.61)	37 (11.78)	1.433 (0.798-2.572)	0.232	104 (25.37)	11 (10.58)	2.963 (0.648-13.537)	0.161
Q4	188 (15.93)	28 (14.89)	2.212 (1.162- 4.214)	0.016	39 (9.51)	4 (10.26)	9.673 (2.620-35.722)	<0.001
χ^2		17.696				—		
p		＜0.001				0.019		

Model 9: Sensitivity analysis was performed after excluding 695 patients with HbA1c≥6.50% or/and HDL-C<1.0 mmol/L, adjusted for gender, age at screening, years, marital status, educational level, job, residential status, annual personal income, family history of coronary heart disease, family history of hypertension, family history of diabetes, smoking status, drinking status, exercise habits, dietary taste, dietary habits, vegetable consumption frequency, fruit consumption frequency, hypertension and overweight/obesity.

Model 10: Sensitivity analysis was performed after excluding 1465 patients with hypertension or/and diabete sor/and dyslipidemia or/and overweight/obesity, adjusted for gender, age at screening, years, marital status, educational level, job, residential status, annual personal income, family history of coronary heart disease, family history of hypertension, family history of diabetes, smoking status, drinking status, exercise habits, dietary taste, dietary habits, vegetable consumption frequency, fruit consumption frequency.

Abbreviations: Q1. Quartile 1; Q2. Quartile 2; Q3. Quartile 3; Q4. Quartile 4. CI. confidence interval; HR. hazard ratio; CVD.cardiovascular disease; HDL-C.high-density lipoprotein cholesterol; HbA1c, hemoglobin A1c

—. Fisher exact test

Supplementary Table 23 Association between HbA1c/HDL-C ratio and cardiovascular disease risk by cox regression analyses among excluded participants.

Variables	β	S.E	Z	*P*	HR (95%*CI*)
HbA1c/HDL-C ratio level					
Q1					1.000 (Reference)
Q2	-0.342	0.762	-0.449	0.654	0.710 (0.160-3.162)
Q3	-0.438	0.756	-0.579	0.562	0.645 (0.147-2.842)
Q4	1.406	0.686	2.049	0.040	4.082 (1.063- 15.667)
Gender					
Males					1.000 (Reference)
Females	1.186	0.594	1.997	0.046	3.273 (1.022-10.481)
Age at screening, years					
60-64					1.000 (Reference)
65-69	0.394	0.476	0.827	0.408	1.482 (0.583-3.768)
70-74	3.172	0.771	4.115	<.001	23.844 (5.264-108.004)
≥75	1.739	0.714	2.436	0.015	5.689 (1.404-23.049)
Marital status					
Separated/divorced/widowed/unmarried					1.000 (Reference)
Married/cohabiting	1.743	1.506	1.157	0.247	5.716 (0.298-109.487)
Educational level					
Primary school and below					1.000 (Reference)
Junior middle school	0.635	0.627	1.013	0.311	1.887 (0.552-6.448)
Technical secondary/high school	1.032	0.704	1.464	0.143	2.806 (0.705-11.160)
College and above	1.210	0.856	1.413	0.158	3.353 (0.626-17.968)
Job					
Others					1.000 (Reference)
Farmers	-0.742	0.541	-1.372	0.170	0.476 (0.165-1.374)
Residential status					
Living alone					1.000 (Reference)
Living only with spouse	-1.241	1.010	-1.228	0.219	0.289 (0.040-2.094)
Living only with children	0.178	1.349	0.132	0.895	1.195 (0.085-16.797)
Living with spouse and children	-1.012	0.964	-1.050	0.294	0.363 (0.055-2.404)
Annual personal income, yuan					
＜4,999					1.000 (Reference)
5,000-9,999	0.509	0.889	0.572	0.567	1.664 (0.291-9.508)
10,000-19,999	-0.590	1.040	-0.567	0.570	0.554 (0.072-4.257)
≥20 000	-0.303	0.850	-0.357	0.721	0.739 (0.140-3.906)
Family history of coronary heart disease					
No					1.000 (Reference)
Yes	0.631	0.624	1.011	0.312	1.879 (0.553-6.382)
Family history of hypertension					
No					1.000 (Reference)
Yes	-0.815	0.520	-1.567	0.117	0.443 (0.160-1.227)
Family history of diabetes					

No					1.000 (Reference)
Yes	-0.506	0.685	-0.738	0.460	0.603 (0.157-2.310)
Hypertension					
No					1.000 (Reference)
Yes	-0.464	0.456	-1.017	0.309	0.629 (0.257-1.537)
Diabetes					
No					1.000 (Reference)
Yes	-0.164	0.555	-0.295	0.768	0.849 (0.286-2.518)
Dyslipidemia					
No					1.000 (Reference)
Yes	0.133	0.441	0.303	0.762	1.143 (0.482-2.712)
Overweight/obesity					
No					1.000 (Reference)
Yes	-0.093	0.447	-0.207	0.836	0.912 (0.379-2.191)
Smoking status					
No					1.000 (Reference)
Yes	1.151	0.641	1.794	0.073	3.160 (0.899-11.107)
Drinking status					
Non-drinker					1.000 (Reference)
Light drinking	0.268	0.594	0.451	0.652	1.308 (0.408-4.192)
Heavy drinking regularly	0.028	0.835	0.033	0.973	1.028 (0.200-5.282)
Exercise habits					
Regular exercise					1.000 (Reference)
Insufficient exercise	0.869	0.519	1.673	0.094	2.384 (0.862-6.595)
Dietary taste					
Moderate					1.000 (Reference)
Light taste	-1.801	1.580	-1.140	0.254	0.165 (0.007-3.651)
Salty taste	-0.255	1.218	-0.209	0.834	0.775 (0.071-8.436)
Dietary habits					
Balanced diet					1.000 (Reference)
Vegetarian-biased diet	-0.118	1.547	-0.076	0.939	0.889 (0.043-18.452)
Meat-biased diet	-0.088	1.605	-0.055	0.956	0.915 (0.039-21.268)
Vegetable consumption frequency					
≤2 days per week					1.000 (Reference)
3-4 days per week	1.170	0.902	1.298	0.194	3.222 (0.550-18.864)
≥5 days per week	1.262	1.016	1.242	0.214	3.534 (0.482-25.911)
Fruit consumption frequency					
≤2 days per week					1.000 (Reference)
3-4 days per week	-2.090	1.074	-1.946	0.052	0.124 (0.015-1.015)
≥5 days per week	-1.187	0.666	-1.783	0.075	0.305 (0.083-1.125)

Abbreviations: Q1. Quartile 1; Q2. Quartile 2; Q3. Quartile 3; Q4. Quartile 4. CI. confidence interval; HR. hazard ratio; HDL-C.high-density lipoprotein cholesterol; HbA1c, hemoglobin A1c
